# Investigation of Anthropogenic and Emerging Contaminants in Sinkholes (Cenotes) of the Great Mayan Aquifer, Yucatán Peninsula

**DOI:** 10.1007/s00244-025-01149-2

**Published:** 2025-09-09

**Authors:** Sarah Kopczynski, Rayna Nolen, David Hala, Fernanda Lases-Hernández, Wendy Escobedo-Hinojosa, Flor Arcega-Cabrera, Ismael Oceguera-Vargas, Antonietta Quigg

**Affiliations:** 1 Department of Marine Biology, Texas A&M University at Galveston, 200 Seawolf Parkway, Galveston, TX 77553 USA; 2 Eurofins Environment Testing North Central, 180 S Van Buren Avenue, Barberton, OH 44203 USA; 3 Department of Ecology and Conservation Biology, Texas A&M University, 534 John Kimbrough Boulevard, College Station, TX 77843 USA; 4 Unidad de Química en Sisal, Facultad de Química, Universidad Nacional Autónoma de México, Puerto de Abrigo Sisal, 97355 Yucatán, México; 5 Department of Oceanography, Texas A&M University, 3146 TAMU, College Station, TX 77843 USA

## Abstract

Karst water bodies are vital groundwater resources particularly vulnerable to pollution. Protecting their water quality requires documenting contaminants traditionally associated with anthropogenic activities (metals, nutrients, and fecal indicator bacteria) as well as emerging contaminants, such as antibiotic-resistant organisms (AROs) and perfluoroalkyl substances (PFAS). This study detected contaminants in karst-associated water bodies on the Yucatán Peninsula, including 10 sinkholes (cenotes) and one submarine groundwater discharge (SGD) site. The concentrations of metals (strontium, cadmium, nickel, lead), nutrients (phosphate, silicate, ammonium, nitrate, and nitrite), and fecal indicator bacteria (fecal coliforms, *Escherichia coli*) were consistent with previous reports, sometimes exceeding recommended standards for groundwater or the protection of aquatic life. This included elevated lead (80.3 µg/L) and nitrate (413 μmol/L) concentrations at two cenotes, and elevated *E. coli* levels (167 – 1800 CFU/100 mL) in five cenotes. Additionally, 34 antibiotic-resistant *E. coli* strains were identified in nine cenotes and most strains were multidrug-resistant. Perfluorooctanesulfonic acid (PFOS) and perfluorohexanoic acid (PFHxA) were also detected in eight cenotes and the SGD, with total PFAS concentrations from 0.68 to 10.71 ng/L. The absence of associations between contaminants and urban cover suggests karst hydrology influences contaminant cycling—stable isotope signatures (δ^18^O, δ^2^H) confirming that most systems are interconnected to regional groundwater flows, that could allow contaminants to travel long distances. The Yucatán Peninsula’s karst is an important freshwater reservoir used for consumption and recreation; the presence of contaminants and the karst’s vulnerability to their spread raises concerns and highlights the need for continued monitoring and conservation.

**Supplementary Information:**

The online version contains supplementary material available at 10.1007/s00244-025-01149-2.

The deterioration of water resources is a global issue, in part driven by anthropogenic contaminants. Polluted runoff from human activities, such as agriculture and industry, along with insufficient wastewater treatment prior to disposal, has significant consequences for aquatic ecosystems worldwide (Freeman et al. 2019). Karst groundwater resources are of particular concern due to their critical role in supplying water to 10–25% of the world’s population (Ford and Williams 2007; Stevanović 2019). Karst landscapes are produced as the dissolution of soluble rock creates a network of interconnected fissures, fractures, and conduits (Ford and Williams 2007). Rainwater rapidly infiltrates these features and permeates the karst soil, effectively bypassing the natural filtration that typically occurs in other less permeable soils. As a result, pollutants in rainwater and runoff are quickly transported into karst groundwater, threatening their water quality (Arcega-Cabrera et al. 2021). Despite their global significance, evaluating water quality in karst is challenging due to their complex and heterogeneous nature. One of the world’s largest coastal karst regions is located on the Yucatán Peninsula, Mexico (Goldscheider et al. 2020) and supports the Great Mayan Aquifer. This freshwater reservoir is the sole source of potable water for the region’s population (Leal-Bautista et al. 2011). Like many karst systems, the Great Mayan Aquifer not only serves as a water resource but also plays a significant role in the cultural and ecological landscape of the Yucatán Peninsula (Bledsoe et al. 2022). Consequently, understanding pollutant stressors to the Great Mayan Aquifer is important regionally and provides applicable insights to other karst ecosystems.

Karst water quality on the Yucatán is commonly assessed through sinkholes (Moreno-Pérez et al. 2021), locally called cenotes, that are considered the surface expression of the Great Mayan Aquifer (Cejudo et al. 2022) and are directly connected to the aquifer by porous rock, cracks, and fractures. Studies have documented various chemical and biological contaminants in cenotes (Moreno-Pérez et al. 2021). Metals, nutrients, and fecal indicator bacteria (e.g., fecal coliforms and *Escherichia coli*) are among the most prevalent in the literature (Arcega-Cabrera et al. 2021, 2023; Moreno-Pérez et al. 2021). All of which have known biological roles, but their biogeochemical cycles can be altered by human activities. When metals and nutrients exceed natural levels, or fecal indicator bacteria enter aquatic ecosystems, they can disrupt ecological stability (Camargo and Alonso 2006; Paruch et al. 2019; Shah 2021). As a result, each is extensively utilized to pinpoint human influences on aquatic ecosystems, including the contamination of karst waters (Long et al. 2012; Pu et al. 2014; Liu et al. 2019; Buckerfield et al. 2020; Camacho-Cruz et al. 2020; Arcega-Cabrera et al. 2023). Therefore, in the context of this study, metals, nutrients, and fecal indicator bacteria will be referred to as ‘anthropogenic’ contaminants. These contaminants remain valuable to study, but incorporating the assessment of emerging contaminants is essential to address evolving threats to water quality.

Contaminants of emerging concern (CECs) are a group of synthetic and naturally occurring chemicals and microorganisms not yet included in existing management protocols but posing potential ecological and human health risks (Feng et al. 2023). CECs have become an important area of research and recent studies have documented the presence of pharmaceuticals (Leal-Bautista et al. 2011; Metcalfe et al. 2011), UV filters (Cooney et al. 2023), microplastics (Mendoza-Olea et al. 2022), and antibiotic-resistant genes (Moore et al. 2020) in cenotes. However, studies on CECs in cenotes remain limited, reflecting a general pattern of sparse investigation of CECs in karst (Lukač Reberski et al. 2022). Two CECs have garnered considerable attention in recent decades, antibiotic-resistant organisms (AROs) and perfluoroalkyl substances (PFAS). AROs, and in general, antibiotic resistance, are a top public health threat (WHO 2019) because contact with AROs spreads antibiotic resistance, reducing the efficacy of necessary therapeutic drugs (O’Flaherty and Cummins 2017). PFAS are synthetic compounds that have been incorporated into commercial and industrial products since the 1950s but have gained recent attention due to their widespread environmental contamination (Rankin et al. 2016; Podder et al. 2021), the presence in biota (Giesy and Kannan 2001; Chen et al. 2021), and the toxicity and persistence of some PFAS constituents (Boudreau et al. 2003; Ankley et al. 2021). Despite concerns, research on AROs and PFAS in karst waters is still limited.

The aim of this study was twofold: (i) assess anthropogenic contaminants (metals, nutrients, and fecal indicator bacteria) and those of emerging concern (AROs and PFAS) in ten cenotes and one submarine groundwater discharge (SGD) site along the Yucatán Peninsula and (ii) investigate relationships between the measured contaminants, urban cover, and other environmental parameters (e.g., δ^18^O, δ^2^H, chlorophyll *a*, etc.). CECs entering a water body can depend on their proximity to human activities, leading us to hypothesize that karst waters adjacent to increased urban cover will contain higher levels of contaminants. In the context of the current study, urban cover estimates the land occupied by human development including residential communities, roadways, and impervious surfaces (Karra et al. 2021). This is particularly relevant to the study area along the ‘Riviera Maya,’ where rapid human development driven by a thriving tourism industry has drastically altered the region’s landscape (Herrera-Silveira et al. 2013). Many cenotes in this region are not only conduits to the Great Mayan Aquifer but are also popular recreation sites used by locals and tourists alike. Therefore, the documentation of contaminants in this region has implications for human health and well-being because of the potential for direct contact and intake of contaminants, underscoring the importance of this research.

## Methods

### Study Area

**Fig. 1:**
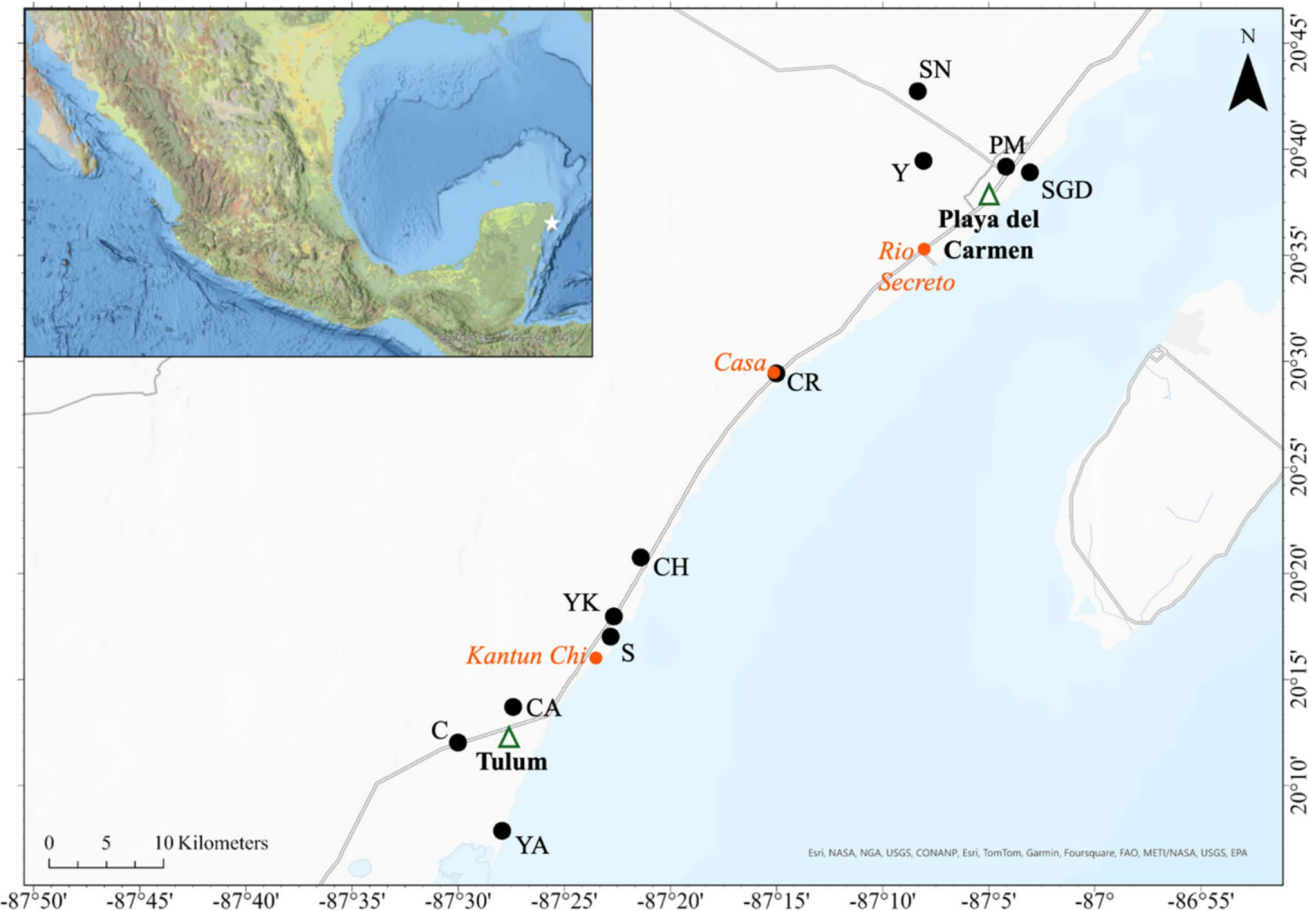
Cenotes and a submarine discharge site (black circles) along the Yucatán Peninsula, Mexico (star), were sampled for anthropogenic and emerging contaminants. Triangles show the locations of the major adjacent cities: Playa del Carmen (north) and Tulum (south). Cenotes (Casa and Kantun Chi) included in the complimentary data provided by the government are highlighted in orange, as well as Rio Secreto the cave system included in the compiled isotopic composition data

Playa del Carmen (population: ~ 300,000) and Tulum (population: ~ 50,000) are rapidly growing communities on the Yucatán Peninsula (Fig. 1). These cities continue to experience growth in their hospitality, retail, and entertainment industries to support tourism, with more than 16 million people visiting them annually (WR 2022). Ten cenotes and one submarine groundwater discharge (SGD) site were sampled across this region in the state of Quintana Roo, Mexico, spanning a geographic transect of ~ 80 km (Fig. 1). Systems were selected based on their human use (Table 1) and varied in their distance to the coast, ranging from < 1 km to over 10 km inland. Anonymized names are given to the systems sampled herein in consideration of the importance of these karst waters to local communities, thereby supporting efforts to protect these economically, ecologically, and culturally significant ecosystems.

**Table 1:** Site characteristics of the sampled cenotes and submarine groundwater discharge site. These sites are either locations for local communities and tourists (herein called recreational) or have limited access because they are cavernous (PM and Y) or closed to the public (S). Urban cover is reported as a percentage, and daily precipitation and air temperature are reported for September 2022 (field campaign)

	Cenote Type	Light Input	Human Access	Urban Cover	Precipitation (mm)	Max and Min Air Temperature	Weather Station
CA	Pit	Low	Recreation	21.7	3.5	23–31 °C	Tulum
CH	Pit	Low	Recreation	11.0	3.5	23–31 °C	Tulum
C	Open	High	Recreation	18.5	3.5	23–31 °C	Tulum
CR	Open	High	Recreation	48.6	0	22–33 °C	Playa del Carmen
PM	Cavernous	Low	Limited	96.2	0	22–33 °C	Playa del Carmen
SN	Open	High	Recreation	0.3	0	22–33 °C	Playa del Carmen
S	Estuarine	High	Limited	1.8	0	22–33 °C	Playa del Carmen
YA	Open	High	Recreation	9.7	0	23–31 °C	Tulum
YK	Open	High	Recreation	1.6	0	23–31 °C	Tulum
Y	Cavernous	Low	Limited	8.8	0	23–31 °C	Tulum
SGD	SGD	High	Recreation	25.5	0	23–31 °C	Tulum

This region has a tropical wet-dry climate based on the Köppen-Geiger climate classification and experiences two main seasons marked by precipitation that are separated by an inter-season drought (Magaña et al. 1999; Peel et al. 2007). The average (1998–2023) rainfall and temperature in September are 175 mm and 28.2 °C in Playa del Carmen (Comisión Nacional del Agua; CONAGUA 2024), and daily precipitation and air temperature during sampling are documented in Table 1.

#### Urban Cover

The Riviera Maya has undergone land-use alterations in recent decades because of increased human development (Herrera-Silveira et al. 2013). To evaluate how this may contribute to contaminant loading, we calculated the area of land used for human infrastructure surrounding each cenote and the SGD site, herein referred to as urban cover. This was evaluated in ESRI ArcGIS Pro (v 3.0) using the global Sentinel − 2 10 m land-use/land-cover time series (v 003) (Karra et al. 2021) trimmed to the sampling month (9/1/2022–10/1/2022). Sentinel-2 evaluates land use based on type (water, trees, flooded vegetation, crops, built area, bare ground, snow/ice, clouds, and rangeland) at a resolution of 10 m. ‘Built area’ is designated as human-made structures, major roads and railways, large homogenous impervious surfaces, industrial buildings, and residential housing.

Built area and total area were calculated within a 500 m radius buffer zone of each sampling site using the ArcGIS Pro Zonal toolset. Urban cover was then calculated by dividing the sum of the built area by the total area within each buffer and reported as a percentage. The effect of land use on water quality is scale dependent (Zhou et al. 2012). We use a 500 m buffer zone because smaller buffer areas are more effective when evaluating the impact of land use on the water quality of specific aquatic ecosystems, particularly when assessing developed land, whereas larger buffer zones are effective for regional water quality management or larger water bodies (Song et al. 2021; Zhang et al. 2021). Current approaches assessing the impact of land use on water quality are not optimized for karst with interconnected regional flows that likely have sources of contamination outside of the buffer zone. Nonetheless, given the many confounding factors in determining an appropriate buffer zone, this approach provides a reasonable first determination of the relationship between urban cover and contaminant loading in the studied systems.

#### Surface Water Sampling

During September 2022 (wet season), the ten cenotes and the SGD were each sampled once between 08:00 and 14:00 h over the course of three days. At each site, a calibrated water quality probe (Hydrolab MS 5, Hach, Loveland, CO) measured in situ temperature, salinity, and pH of surface waters (top 1 m). Polyethylene terephthalate water bottles washed 3 × with site water were then used to collect 4 L. For the submarine groundwater discharge site, a modified sampling protocol was used where water quality measurements and the collected surface water was taken directly from the discharge plume. The 4 L collected was immediately placed on ice in a covered cooler until sample storage and processing (within 8 h) where aliquots were subsampled for nutrient, chlorophyll *a*, bacterial, and PFAS analyses. For metals and isotope hydrology, surface water was collected as described above, but using a separate Nalgene bottle filled without headspace and stored at 25 °C until analysis.

#### Metals and Ions

Water samples were acidified with ACS grade nitric acid (J.T. Baker, Phillipsburg, NJ, USA) to a pH of 2 and analyzed following Arcega-Cabrera and Fargher (2016). Strontium (Sr) was measured using a PerkinElmer (PerkinElmer, Waltham, MA, USA) AAnalyst 800 Atomic Absorption Spectrometer on air-acetylene flame mode, while cadmium (Cd), nickel (Ni), and lead (Pb) were analyzed on a PerkinElmer PinAAcle900 Atomic Absorption Spectrometer (PerkinElmer, Waltham, MA, USA) coupled to a graphite furnace. Both analyses were corrected using a deionized water blank. Instrument detection limits for these elements are reported in Supplemental Table 1. All other metals and ions were measured according to Arcega-Cabrera et al. (2023) (see Supplemental Table 2 for reagents and standards).

#### Nutrients and Chlorophyll a

An aliquot of surface water (100 mL) from each site was stored at − 20 °C until processing. Thawed samples were immediately filtered on Whatman glass fiber filters (0.7 µm), and the filters and filtrate stored again at  − 20 °C until analysis, at which time the filters and filtrate were thawed and immediately processed using their respective methods. Dissolved nutrients were analyzed by the Texas A&M University Geochemical and Environmental Research Group using a Lachat QuikChem AE autoanalyzer to determine phosphate, silicate, ammonium, nitrate, and nitrite. All dissolved nutrients are expressed as µmol/L (see Supplemental Table 3 for concentrations reported in mg/L), except the ratio of dissolved inorganic nitrogen to phosphorus (DIN/P). DIN was calculated by summing ammonium, nitrate, and nitrite. The filter was extracted for chlorophyll (chl) *a* based on the U.S. EPA method 445.0 (Arar and Collins 1997) and used *Anacytis nodulans* chlorophyll *a* powder (Sigma-Aldrich, St. Louis, MO, USA) as the calibration standard. Briefly, chl *a* was extracted by homogenizing filters with 90% acetone and filter material was removed via centrifugation. Samples were measured on a calibrated 10 AU Turner Designs fluorometer (Turner Designs, San Jose, CA, USA) before and after acidification with 10% hydrochloric acid. Chl *a* fluorescence was blank (Milli-Q) corrected and reported as µg/L.

#### Bacterial Enumeration

An aliquot (100 mL) from each site was filtered through PC membrane filters (25 mm, 0.2 µm, Isopore™). Sterile forceps were used to transfer the filters into cryotubes containing 1 mL of sterile 70% glycerol in peptone water (0.1%). Filters were immediately preserved in liquid nitrogen until processing. Samples were then thawed and transferred into 15 mL falcon tubes containing 100 mg of sterile mini-glass beads (0.5 mm). Bacteria adhered to the filters were suspended by vortexing (5 min) and then transferred into 2 mL Eppendorf tubes. Filters underwent a secondary rinse of 1 mL of peptone diluent (0.1%) and were vortexed to ensure all adhered bacteria were collected. The obtained wash yielded 2 mL of resuspended biomass and was serially diluted in peptone diluent (0.1%), up to 1 × 10^−10^. 100 μL of each dilution was inoculated with glass beads (5 mm) in three different media (see Supplemental Table 4 for supplier information): (1) plate count agar, (2) violet, red bile agar, and (3) ECD Chromoselect agar with 4-methylumbelliferyl-ß-D-glucuronide (MUG), which quantified heterotrophic aerobic bacteria, coliforms/fecal coliforms, and *Escherichia coli*, respectively (Clesceri et al. 1998; Feng and Hartman 1982; Greenberg et al. 1992). Plates were incubated at various temperatures and times (Supplemental Table 4), and those containing countable isolated colonies were used to determine the colony forming units (CFU) per 100 mL of the sampled water. All other reagents and the sterile beads for bacterial isolation were obtained from Sigma-Aldrich (St. Louis, MO, USA).

#### Antibiotic-Resistant Organisms (AROs)

While enumerating bacteria growing on ECD Chromoselect Agar with MUG, *E. coli* strains*,* a biomarker of anthropogenic influence, were phenotypically identified by their distinctive blue coloration and fluorescence under UV light. Several isolated colonies were randomly selected and transferred to Luria–Bertani (LB) agar media. Susceptibility testing was performed using the Kirby Bauer disk agar diffusion method in accordance with the Clinical and Laboratory Standards Institute (CLSI) guidelines (Weinstein 2020). Eight antimicrobial agents from Sigma-Aldrich (St. Louis, MO, USA) were tested: the macrolides, erythromycin (10 μg/disk), and clarithromycin (2 μg/disk); the beta-lactams, penicillin G (10 μg/disk), and ceftibuten (1 μg/disk); the fluoroquinolone, levofloxacin (5 μg/disk); the phenicol, chloramphenicol (25 μg/disk); the sulfonamide, sulfamethoxazole (25 μg/disk); and the tetracycline, tetracycline (10 μg/disk). Test results were interpreted according to the M100 criteria of the CLSI (Weinstein 2020). The model bacterium *Enterococcus faecalis* ATCC 29212 (American Type Culture Collection, Manassas, VA, USA) was used as a control strain in the disk agar diffusion test.

#### PFAS Extraction and Quantification

Duplicate water samples (1 L) from each site were spiked with internal standards 13C4-perfluorooctansulfonate (13C4-mPFOS, Wellington Labs, Guelph, ON, CA) and 13C8-perfluorooctanoic acid (13C8-PFOA, Cambridge Isotope Labs, Tewksbury, MA, USA) at 50 ng/mL (final concentration). Samples were immediately processed using solid phase extraction (SPE) on Bond Elut 500 mg/6 mL Agilent Technologies (Santa Clara, CA, USA) wax cartridges. Each SPE was conditioned with 15 mL methanol and 28 mL deionized water to maintain the solvation of the cartridge sorbent bed. Glass wool (Sigma-Aldrich, St. Louis, MO, USA) removed particulate matter as samples were extracted through the SPE cartridges under vacuum (10 mL/min). Sample bottles were rinsed twice (7.5 mL) with deionized water and following extraction, and the cartridges were dried under vacuum for 5 min. Cartridges were stored at room temperature and transported back to Texas A&M University at Galveston. The cartridges were eluted in methanol, dried down under a gentle stream of nitrogen in a water bath (60 °C), and the extracted PFAS reconstituted in methanol. Extraction steps were performed following the methods outlined in U.S. EPA Method 537.1 with few modifications, and in accordance with recommended hold times (Shoemaker et al. 2008). All samples were analyzed on an Agilent 1260 ultra-high-performance liquid chromatographer with triple-quad 6420 mass detector (Agilent, Santa Clara, CA, USA), and all solvents were high performance liquid chromatography grade (Sigma-Aldrich, St. Louis, MO, USA).

LC–MS/MS analysis was conducted based on published methods (Nolen et al. 2022, 2024) for 12 U.S. EPA priority PFAS detailed in Supplemental Table 5. A 9-point standard curve (0.78 to 200 ng/mL) was run for each PFAS and the internal standards (50 ng/mL final concentration). Compound separation was performed at a flow rate of 0.4 mL/minute on an Agilent ZORBAX Eclipse Plus C18 RRHD column (3.0 × 50 mm, 1.8 μm; Agilent, Santa Clara, CA, USA). The liquid mobile phase comprised: Milli-Q water (A) and methanol (B), both containing 5 mM ammonium acetate (Sigma-Aldrich, St. Louis, MO, USA). The mobile phase gradient is detailed in Supplemental Table 6. Multiple reaction monitoring (MRM) was used to detect the precursor and product ions for all compounds under negative electrospray ionization (ESI-) mode with nitrogen as the desolvation gas (Supplemental Table 5). The limit of detection (LOD) was set to the lowest calibration point with an accuracy of ≥ 70% and precision of ≤ 20% (Supplemental Table 5. All PFAS standards were purchased from Sigma-Aldrich (St. Louis, MO, USA).

For quality assurance and quality control measures, we optimized PFAS extraction efficiency with Milli-Q water in polyethylene bottles (*n* = 3) spiked with a representative mixed standard of all listed compounds and quantified each compounds percent recovery (Supplemental Table 5). A method blank (Milli-Q water) was extracted and analyzed alongside the samples for blank correction to remove PFAS interference during the extraction process. Levels above the limit of detection are reported. Supplemental Table 7 details all average concentrations (ng/L) and standard deviations for duplicates, but only PFAS detected in both site replicates were included in the statistical analysis.

#### Stable Isotope Analysis

Samples were equilibrated with ultra-high purity 0.3% CO_2_/He (Praxair, Mérida, YU, MX) for 20 hours, then with ultra-high purity 2% H_2_/He mixture (Praxair, Mérida, YU, MX) for two hours at 25 °C. Measurement of isotopes was conducted at Laboratorio de Análisis de Isótopos Estables (Yucatán, MX) using a Gasbench II sample preparation system coupled with a Delta-V PLUS isotope ratio mass spectrometer (Thermo Fisher Scientific, Waltham, MA, USA). Samples were analyzed alongside Vienna Standard Mean Ocean Water 2 and Standard Light Antarctic Precipitation 2 (VSMOW2 and SLAP2; International Atomic Energy Agency, Vienna, AT), as well as two in-house standards for quality control (− 10.4 and − 6.9 % δ^18^O; − 74.6 and − 46.2% δ^2^H), consisting of rainwater normalized against VSMOW2, SLAP2, and GRESP (Greenland Summit Precipitation; International Atomic Energy Agency, Vienna, AT). Long-term measurement uncertainties were 0.2% for δ^18^O and 2% for δ^2^H. Results are reported as the conventional delta notation (δ^18^O and δ^2^H), representing the relative deviation of the heavy-to-light isotope ratio of the sample compared to VSMOW expressed in per mil (%) (Coplen 1996).

The deuterium excess (d-excess) is a function of δ^18^O and δ^2^H used to understand evaporation of water from its source and was calculated as *d* = δ^2^H – 8.17 δ^18^O. This formula incorporates the slope (8.17) of the Local Meteoric Water Line for Quintana Roo (Lases-Hernandez et al. 2019), which is a better representative of regional isotopic rainfall data (Clark and Fritz 2013) compared to the Global Meteoric Water Line (GMWL)—a linear relationship between δ^18^O and δ^2^H used to understand the conditions in which meteoric waters evaporate and condense globally (Craig 1961; Rozanski et al. 1992). Furthermore, we compiled regional groundwater (Socki et al. 2002; Wassenaar et al. 2009; Hodell et al. 2012; Lases-Hernandez 2013; Evans et al. 2018; Lases-Hernandez et al. 2019, 2020) and seawater (Socki et al. 2002; Perry et al. 2003; Lases-Hernandez 2013; Haukebo 2014) isotopic data from studies across the Yucatán and Quintana Roo States to place the δ^18^O and δ^2^H of our systems into a regional context. Studies were included if published between 1994 and 2019 and if they reported more than one site and/or more than one sampling date. From the compiled data, the average isotope composition of Rio Secreto (20.62° N,  − 87.15° W; Fig. 1)—a semi-flooded cave in Playa del Carmen—is reported from a long-term monitoring study and included because it represents the aquifer saturated zone (Lases-Hernandez et al. 2019).

#### Complimentary Data

We acquired additional data (2021–2023) pertinent to cenotes in northern Quintana Roo through Mexico’s Federal Law of Transparency and Public Information Access (INAI 2015). This included monthly *E. coli* levels monitored by the Mexican Federal Commission for the Protection against Sanitary Risk (COFEPRIS), and information related to Quintana Roo’s state tourism (i.e., hotel occupancy rates for Riviera Maya, and the number of international passengers arriving and departing Quintana Roo airports) provided by the Quintana Roo Tourism Secretary (SEDETUR). COFEPRIS monitors several cenotes in Quintana Roo including five cenotes close to, or overlapping with, the sites of interest: CA, C, CR, Casa, and Kantun Chi (Fig. 1). *E. coli* concentrations reported by COFEPRIS are analyzed using their standard procedure (CCAYAC-M-004/11) and reported as NMP/100 mL which is equivalent to CFU/100 mL.

#### Statistical Analysis

All statistical analyses were conducted in R software version 4.2.2 (R Core Team 2022). Kendall’s rank correlation coefficients between contaminants and the physical, chemical, and biological parameters were calculated using the Hmisc package (v 5.1.0; Harrell 2023). Correlations between parameters were conducted twice incorporating data (i) from all sample sites (n = 11) and (ii) from a subset that included the open, pit, and estuarine cenotes (n = 8; Table 1). Correlation coefficients were tested for their statistical significance using an alpha of 0.05, and the correlation matrices were visualized with corrplot (v 0.92; Wei and Simko 2021). Kendall’s rank correlation was performed as an alternative to Pearson’s product–moment correlation because it is an effective, robust method for nonlinear data (Puth et al. 2015) and was appropriate for the sample size (Bujang 2024). When describing the results of correlations between contaminants, urban cover, and other measured environmental parameters (e.g., δ^18^O, δ^2^H, chlorophyll *a*, etc.), we focus our findings on the subset that excluded cavernous cenotes (PM and Y) and the SGD because these systems vary greatly in their hydrology compared to open, pit, and estuarine cenotes. Additional R packages used in the work include ggplot2 (v 3.4.4; Wickham 2016), ggbreak (v 0.1.1; Xu et al. 2021a, b), and RColorBrewer (v 1.1.3; Neuwirth 2022).

## Results

Each cenote was characterized as open, pit, estuarine, or cavernous, and physical characteristics for each site are documented in Table 1. No significant rainfall occurred during sampling (Table 1), but CONAGUA’s meteorological station in Playa del Carmen reported 53 mm of rain on the day prior to the sampling campaign (CONAGUA 2024). Surrounding urban cover was highest at the SGD site (25.5%) and the cenotes, CR (48.6%) and PM (96.2%). The urban cover at all other cenotes was below 25%, and the lowest urban cover (0.3%) was observed at cenote SN (Table 1).

**Fig. 2:**
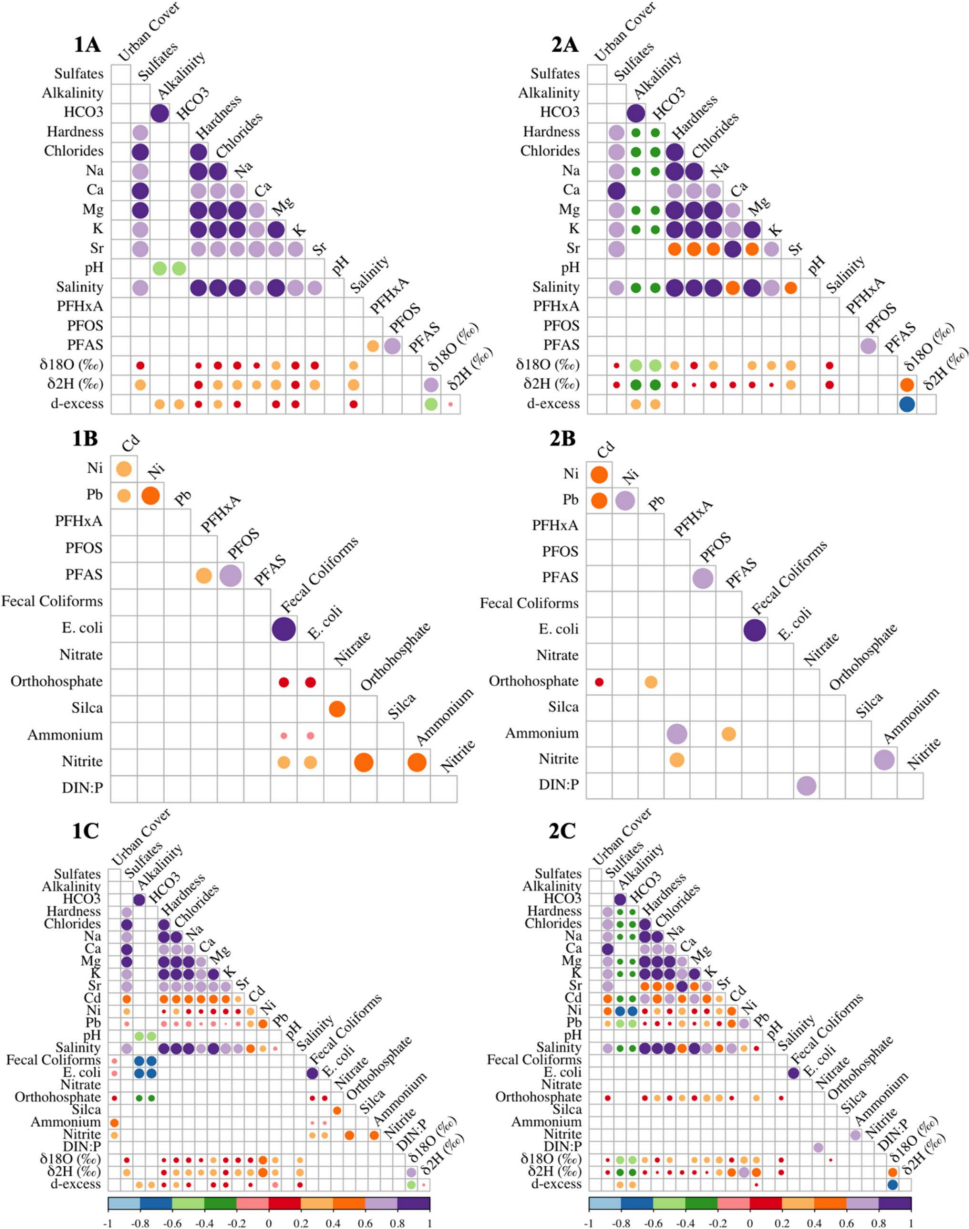
Significant (*p* < 0.05) correlations between parameters calculated using Kendall’s rank correlation coefficients. Correlation of all systems (1) and a subset (*n* = 8) of the pit, open, and estuarine cenotes (2) divided into PFAS vs. environmental parameters (**a**), PFAS vs. legacy contaminants (**b**), and legacy contaminants vs environmental parameters (**c**). Red, orange, and purple coloring represent positive correlation between parameters, whereas blue, green, and pink represent negative correlations. The parameter, chl *a*, did not significantly correlate with another parameter and was removed from the visual. The written results and discussion focus on the correlations documented in panel 2

In situ water temperature (25.5—28.7 ºC) and pH (6.94—7.72) were relatively similar between cenotes, whereas salinity (0.53—22.58 ppt) was more variable (Table 2). The lowest and highest salinity observed at cenotes Y and S, respectively. Alkalinity, bicarbonate (HCO_3_^−^), and hardness ranged from 221 to 364 CaCO_3_ mg/L, 270–445 mg/L, and 345—3425 CaCO_3_ mg/L, respectively. Sulfates and chlorides were lowest at Y and highest at S and reported in ranges as follows: 25.8–504 mg/L and 336–13,056 mg/L (Table 2). Significant correlations were observed between many of these water chemistry parameters (Fig. 2); for instance, salinity was positively correlated with sulfates, hardness, and chlorides, whereas alkalinity was negatively correlated with these same parameters.

**Table 2:** Physical and chemical characteristics of each site. < LOD stands for below the limit of detection, and the dash (-) represents no data at that site (YA) because temperature could not be taken at the time of sampling

	CA	CH	C	CR	PM	SN	S	YA	YK	Y	SGD
Temperature (°C)	27.1	25.5	28.2	25.5	26.7	28.7	28.1	–	25.9	25.8	27.5
pH	7.14	7.37	6.94	7.67	7.63	7.72	7.68	7.30	7.02	7.47	7.24
Salinity (ppt)	1.30	2.23	1.93	2.77	1.39	0.90	22.58	5.37	2.21	0.53	3.35
Alkalinity (CaCO3 mg/L)	355	338	364	351	221	279	228	343	348	248	359
[HCO3-] (mg/L)	433	413	445	428	270	341	278	418	425	302	438
Hardness (CaCO3 mg/L)	573	587	582	714	392	345	3425	1051	687	377	679
Sulfates (mg/L)	154	156	132	148	97	100	504	320	176	25.8	210
Chlorides (mg/L)	1211	1308	1130	2288	878	511	13,056	3903	2126	336	2934
Mg (mg/L)	80.9	89.9	81.0	125.5	56.4	44.7	794.8	270.7	104.7	28.2	136.8
Na (mg/L)	710	740	714	1323	519	329	7736	2426	880	209	1231
Ca (mg/L)	223	216	207	213	159	168	323	248	225	176	224
K (mg/L)	36.89	34.71	30.89	44.02	32.49	24.73	289.8	76.73	39.95	25.49	50.85
Sr (mg/L)	2.40	2.16	2.33	2.02	1.32	0.80	8.64	3.71	2.60	0.79	2.37
Cd (µg/L)	0.03	0.04	0.03	0.04	< LOD	0.03	0.35	0.08	0.02	0.07	0.06
Ni (µg/L)	1.41	2.04	< LOD	< LOD	4.30	3.60	15.72	7.40	< LOD	2.34	8.40
Pb (µg/L)	0.56	0.42	0.36	0.37	1.09	0.56	80.3	0.44	0.13	1.20	0.48
δ^18^O (% VSMOW)	− 4.6	− 4.7	− 4.1	− 4.5	− 4.1	− 3.1	− 1.1	− 3.6	− 4.4	− 4.4	− 4.1
δ^2^H (% VSMOW)	− 23.7	− 23.7	− 23.1	− 25.6	− 23.5	− 19.2	− 6.4	− 17.4	− 27.2	− 26	− 21.8
d-excess	13.9	14.7	10.4	11.2	10.0	6.1	2.6	12.0	8.7	9.9	11.7

### Metals and Ions

The heavy metals, cadmium (Cd), nickel (Ni), and lead (Pb) were documented in concentration ranges as follows when detected: 0.02–0.35 µg/L Cd, 1.41–15.72 µg/L Ni, and 0.13–80.3 µg/L Pb (Table 2). The other metals (magnesium, sodium, calcium, potassium, and strontium) were detected in concentrations from 28.2 to 794.8 Mg mg/L, 209–7736 Na mg/L, 159–323 Ca mg/L, 24.73–289.80 K mg/L, and 0.79–8.64 Sr mg/L (Table 2). The highest level of all metals were reported in cenote S, and the concentration of lead (80.3 µg/L) at this cenote exceeded levels that may damage habitats or early stages of biota in groundwater and freshwater (Buchman 2008). All metals were significantly (*p* < 0.05) positively correlated to one another (Fig. 2); Mg, Na, Ca, K, and Sr also significantly correlated with sulfates, hardness, and chlorides (positive relationship), and with alkalinity and HCO₃⁻ (negative relationship).

### Nutrients and Chlorophyll a

Dissolved nutrient and chl *a* concentrations were site-specific (Table 3). The dissolved nutrient ranges were as follows: phosphate (0.10 – 1.04 μmol/L), silicate (20.39 – 49 μmol/L), ammonium (1.08 – 102 μmol/L), nitrate (2.9—413 μmol/L), and nitrite (0.16 – 6.14 μmol/L) (Table 3). Silicate and nitrate were highest at cenote Y; nitrate exceeding guidelines for the protection of aquatic life (13 mg/L nitrate, equivalent to 210 µmol/L; CCME 2012). The highest concentrations of phosphate, nitrite, and ammonium were observed at cenote PM. All DIN/P ratios were above the Redfield ratio (16:1; Redfield 1934). Nutrient correlations at open, pit, and estuarine cenotes revealed a significant positive relationship between DIN/P and nitrate (Kendall’s tau = 0.64), and ammonium and nitrite (Kendall’s tau = 0.71).

**Table 3:** Nutrient concentrations (µmol/L, μM) and chlorophyll *a* (chl *a* μg/L). Below the detection limit is noted as < LOD. For nutrient concentrations reported in mg/L, see Supplemental Table 3

	Phosphate	Silicate	Ammonium	Nitrate	Nitrite	DIN/P	chl *a*
CA	0.32	26.64	13.7	21.1	3.52	120	< LOD
CH	0.12	40.06	1.46	79.6	0.36	679	< LOD
C	0.25	24.05	2.90	114	1.15	473	0.54
CR	0.25	33.78	2.20	90.6	1.00	375	< LOD
PM	1.04	25.44	102	22.5	6.14	125	< LOD
SN	0.16	22.90	1.08	2.9	0.16	26	6.82
S	0.54	38.85	3.57	27.0	0.60	58	0.72
YA	0.18	36.22	2.94	63.1	1.68	376	5.08
YK	0.20	34.92	2.08	76.8	0.80	398	9.28
Y	0.60	49.00	7.90	413	2.40	705	< LOD
SGD	0.10	20.39	69.2	3.1	0.24	725	0.31

Chl *a* was detected at five cenotes and the SGD site (Table 3) in concentrations from 0.31 µg/L (SGD) to 9.28 µg/L (cenote YK). Each of these systems is designated as an open cenote or SGD and has light input (Table 1); the only open cenote where chl *a* was not detected was CR. Chl *a* was not observed to correlate with any other parameter (Fig. 2).

### Bacteria

**Fig. 3:**
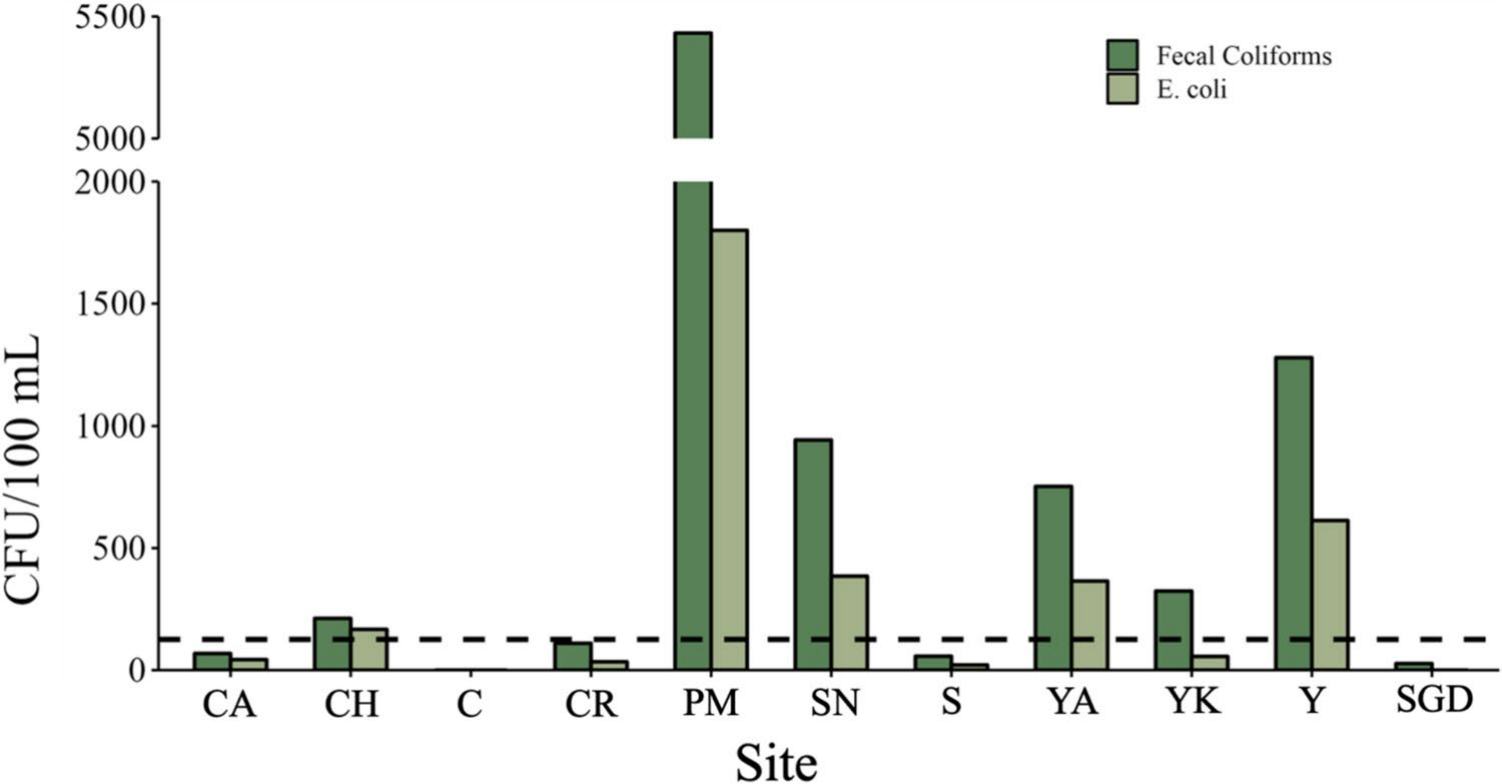
Fecal coliform and *E. coli* concentrations expressed as colony forming units per 100 mL (CFU/100 mL). The dashed line represents the advisory concentration (126 CFU/100 mL) for *E. coli* in recreational water bodies set by the U.S. EPA

Fecal coliforms (27–5,432 CFU/100 mL) were detected in all systems except cenote C, and *E. coli* (21–1,800 CFU/100 mL) in all systems except cenote C and the SGD site (Fig. 3, Supplemental Table 8). The highest abundances of both were observed in cenote PM. *E. coli* levels surpassed the U.S. EPA advisory concentration (126 CFU/100 mL) for recreational waters in five cenotes (U.S. EPA 2012), three of which are used for recreation (Table 1). We compare *E. coli* concentrations to the U.S. EPA advisory level because there is no specific legislation for inland recreational waters in Mexico (Arcega-Cabrera et al. 2023). However, based on the Mexican standard for beach water quality (200 CFU/100 mL E. coli), four cenotes (PM, SN, YA, and Y) contained *E. coli* concentrations exceeding this standard (NMX-AA-120-SCFI-2016). Bacterial concentrations were not significantly (*p* < 0.05) correlated with any other parameters when evaluating open, pit, and estuarine cenotes.

### Antibiotic-Resistant Organisms (AROs)

**Fig. 4:**
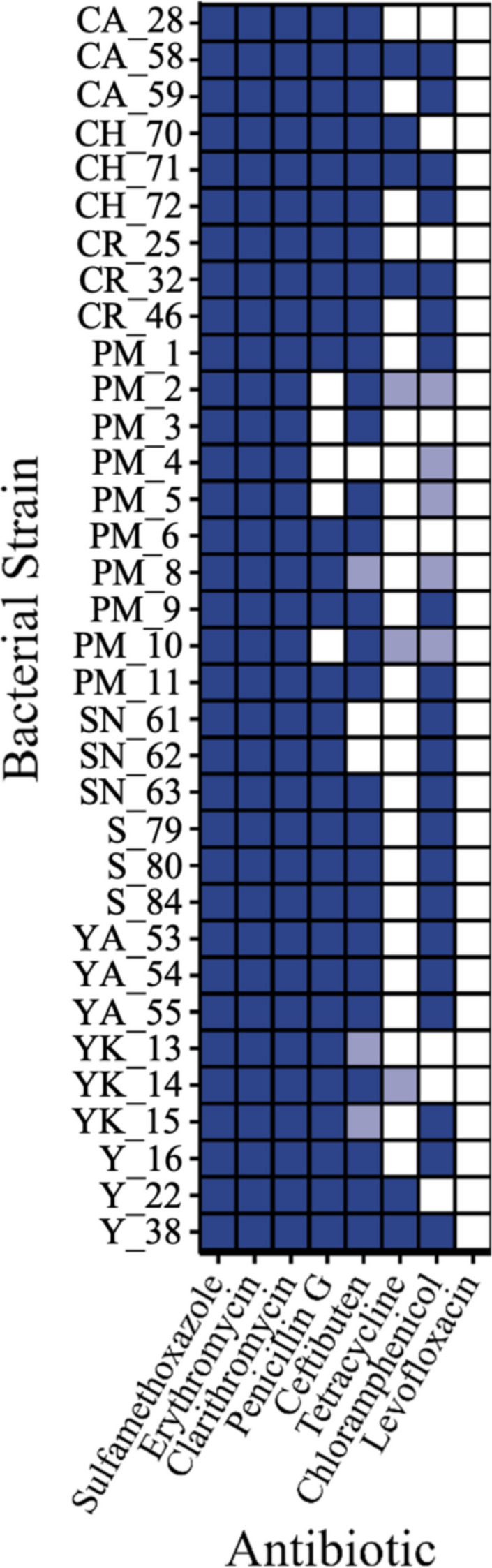
Bacterial strain resistance to antibiotics. Dark blue, light blue, and white indicate that the strain is resistant, slightly sensitive, and sensitive, respectively. Strains are labeled based on their collection site: CA, CH, CR, PM, SN, S, YA, YK, and Y. No strains were isolated from cenote C or the SGD because of the limited *E. coli* measured at those sites

Antibiotic-resistant *E. coli* were detected at nine cenotes (CA, CH, CR, PM, SN, S, YA, YK, and Y) (Fig. 4), and no antibiotic-resistant *E. coli* were detected at cenote C or the SGD. A total of 34 bacterial strains were isolated from the cenotes. Three strains were isolated from each, except for cenote PM in which 10 strains were isolated. All 34 strains were resistant to sulfamethoxazole, erythromycin, and clarithromycin, while 31 were resistant or slightly sensitive to ceftibuten; 29 to penicillin; 26 to chloramphenicol; and 9 to tetracycline. Levofloxacin was the only antibiotic tested that resulted in no growth of any strain. Multidrug resistance was observed for all strains, and 27 of the 34 were resistant to ≥ 5 antibiotics.

### Perfluorinated Alkylated Substances (PFAS)

**Fig. 5:**
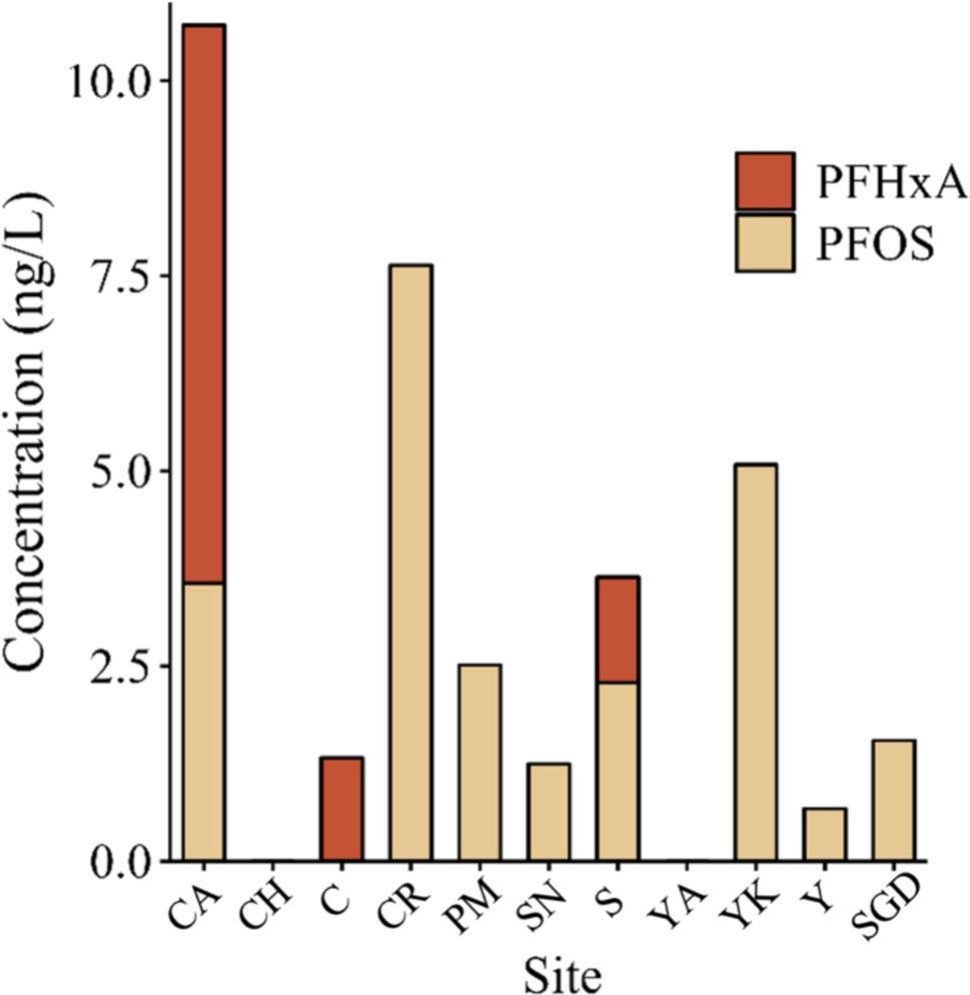
PFOS (perfluorooctanesulfonic acid) and PFHxA (perfluorohexanoic acid) concentrations in ng/L. Standard deviation for duplicates is reported in Supplemental Table 7. PFAS were below the limit of detection at two cenotes (CH and YA)

Nine of eleven sites had detectable levels of at least one PFAS and sum concentrations (Fig. 5; Supplemental Table 7) ranged from 0.68 (± 0.00; cenote Y) to 10.71 ng/L (± 3.65; cenote CA). Perfluorooctanesulfonic acid (PFOS) was more frequently detected than perfluorohexanoic acid (PFHxA); the latter detected in three cenotes (CA, C, and S) (Fig. 5). Other PFAS (PFBS, PFHxS, and PFDoA) compounds were detected at some sites, but only in one duplicate, and thus, they are reported in Supplemental Table 7 and were not used in further analysis. When present, PFHxA was positively correlated to ammonium (Kendall’s tau = 0.71) and to nitrite (Kendall’s tau = 0.36) (Fig. 2).

### Isotope Hydrology

Stable isotope composition for oxygen (δ^18^O) and hydrogen (δ^2^H) ranged from − 4.6% to − 1.1% and − 27.2% to − 6.4%, respectively (Table 2), with the isotope signature at the SGD (-4.10 δ^18^O, -21.8 δ^2^H) aligning directly with the Local Meteoric Water Line. D-excess was lowest at cenote S (2.6) and highest at cenote CH (14.4), the majority (*n* = 8) having a d-excess between 9.9 and 14.7 (Table 2). All isotope parameters were significantly correlated to alkalinity and HCO_3_^−^, a negative relationship observed with δ^18^O (Kendall’s tau = 0.43) and δ^2^H (Kendall’s tau = 0.33), and a positive with d-excess (Kendall’s tau = 0.29) (Fig. 2).

**Fig. 6:**
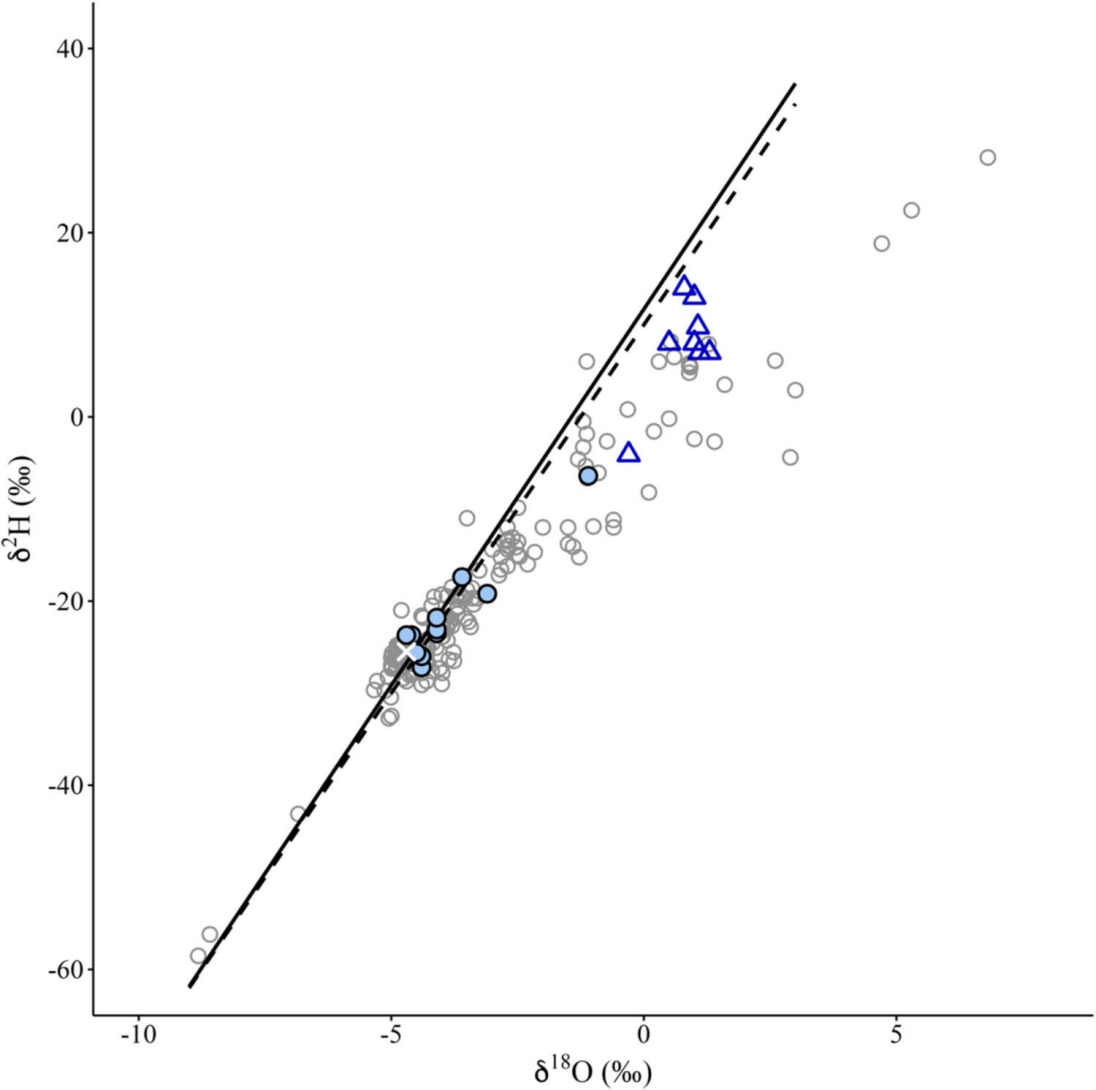
Isotope composition of waters from this study (blue circles) compared to the Global Meteoric Water Line (δ^2^H = δ^18^O 8 + 10, dashed black line; Craig 1961) and the Local Meteoric Water Line (δ^2^H = δ^18^O 8.17 + 11.698, solid black line; Lases-Hernández et al. 2019). The sites are compiled with the δ^18^O and δ^2^H of groundwater (gray circles, *n* = 212) and seawater (blue triangles, *n* = 8) on the Yucatán Peninsula (see methods), as well as the average δ^18^O (-4.7 ± 0.1%) and δ.^2^H (− 25.5 ± 0.9%) composition for the aquifer saturated zone of the Rio Secreto cave system (white cross, *n* = 75; Lases-Hernández et al. 2019, 2020)

Based on data from the compiled literature, most isotopic compositions of Yucatán Peninsula groundwater ranged between − 6 % and − 3% for δ^18^O (frequency > 70%) and − 15% to -30% for δ^2^H (frequency > 70%) (Fig. 6), whereas seawater is reported from − 0.30 to 1.30 δ^18^O and − 4.10 to 14.00 δ^2^H. All sites fall within these common ranges for Yucatán Peninsula groundwater except for cenote S, which had 1.1% δ^18^O and − 6.4% δ^2^H. Four cenotes (CA, CH, CR, Y) had δ^18^O and δ^2^H similar to Rio Secreto’s average isotope signature (− 4.7 ± 0.1% δ^18^O, -25.5 ± 0.9% δ^2^H), varying by less than ± 0.3% δ^18^O and − 1.8% δ^2^H.

### Complimentary Data

Cenote CA, C, CR, Casa, and Kantun Chi were reported to have higher concentrations than the U.S. EPA advisory *E. coli* levels (126 CFU/100 mL) during multiple months of the COFEPRIS 36-month reporting period (Supplemental Fig. 1); 18, 16, 20, 14, and 17 reports (one report per month when available) above the advisory limit in the 36-month period, respectively. Hotel occupancy rates in Riviera Maya ranged from 23 to 87%, and the number of international passengers arriving and departing Quintana Roo airports ranged from 564,485 to 2,120,606 per month in 2021 to 2023. Although the lowest occupancy rates and number of passengers were observed in 2021 when tourism was still recovering post-COVID-19, and the numbers fluctuated with seasons, the tourism indicators increased from 2021 to 2023 (Supplemental Fig. 2).

## Discussion

This study provides a snapshot of water quality and contamination by several anthropogenic and emerging contaminants in cenotes and a submarine groundwater discharge site along the Riviera Maya on the Yucatán Peninsula. We document contamination in most of the studied systems which emphasizes their vulnerability to anthropogenic pollution, with greater contamination often observed at cenotes with high urban cover (e.g., cenote PM) or recreational access (e.g., cenote CA). However, contaminants did not significantly correlate with urban cover as might be expected in non karst ecosystems. The measured isotope signatures suggest that the systems are highly interconnected and indicate that the complexity of water movement in the karst may complicate associating land use with pollutant loading, a common finding when investigating contaminants in karst (Herrera-Silveira et al. 2013; Padilla and Vesper 2018; Arcega‐Cabrera et al. 2021, 2023). However, what remains clear is that continued urban and economic growth fueled by increasing tourism (Supplemental Fig. 2), without improved wastewater management and infrastructure will lead to degraded cenote water quality, with negative consequences on their ecological and social stability. The studied systems are representatives of the region’s karst landscape, making these insights valuable in the context of contaminant loading and fate in coastal karst ecosystems.

### Anthropogenic Contaminants

While many metals have essential biological roles, excess concentrations beyond what can be biologically processed can pollute ecosystems (Shah 2021). The concentrations documented here of metals, including heavy metals, were consistent with previous studies (Arcega-Cabrera et al. 2021, 2023), as well as their interrelationships with other measures (e.g., salinity) (Pérez-Ceballos et al. 2012; Arcega-Cabrera et al. 2023). For instance, we observed positive correlations between magnesium, sodium, calcium, potassium, strontium, sulfates, chlorides, and salinity. This pattern is consistent with prior work (Pérez-Ceballos et al. 2012) and could be attributed to saltwater encroachment (Perry et al. 2002), a process particularly relevant to coastal cenotes. Given that some of the studied systems are very close to the coast (e.g., cenote YA) or directly influenced by the ocean (e.g., cenote S), it is unsurprising that we observed correlations between these measures. One cenote (S) had noticeably higher concentrations of the heavy metal lead (80.3 μg/L) compared to others (0.13–1.20 μg/L), exceeding values (65 μg/L) thought to damage habitats or early stages of biota in groundwater and freshwater (Buchman 2008). This suggests that this cenote (S) could be receiving an input of metals, likely from industrial or agricultural activities. But given its low urban cover (1.8%), metal input may originate far from it, as groundwater in karst can travel significant distances or move from inland along regional flow paths (Bauer-Gottwein et al. 2011) to discharge at this estuarine cenote.

Human activities significantly alter global nutrient cycles (Smith et al. 1999; Camargo and Alonso 2006) leading to the frequent inclusion of nutrient analyses in studies identifying human influence on aquatic environments (Biggs et al. 2004; Bricker et al. 2014; Nazeer et al. 2016). The nutrient concentrations reported herein are comparable to previous assessments of cenotes (Schmitter-Soto et al. 2002; Leal-Bautista et al. 2011), though concentrations of some nutrients indicate human-derived input. Nitrate and ammonium are particularly useful tracers in this context; these forms of nitrogen are elevated in aquatic ecosystems inundated by agricultural runoff or untreated wastewater, respectively (Domingues et al. 2011). Nitrate levels above 4.0 mg/L (64.5 µmol/L) often indicate anthropogenic inputs (CCME 2012) and five cenotes (CH, C, CR, YK, and Y) exceed this level, with cenote Y (25.6 mg/L, 413 µmol/L) surpassing suggested guidelines for the protection of aquatic life (13 mg/L; CCME 2012). These levels could result from agricultural runoff, or at cenote Y, deforestation could be a contributing factor because this system was recently discovered in February 2022 (Cenotes Urbanos Speleological Group) due to deforestation for the construction of the Mayan train project (Hernández-Stefanoni et al. 2024). Deforestation increases nutrient leaching which can facilitate the mobilization of nitrate to nearby water bodies (Rusinga et al. 2008; Robinson et al. 2022).

Regarding ammonium, Camacho-Cruz et al. (2020) investigated cenotes in the same region and reported a maximum concentration of 4.6 μmol/L (0.08 mg/L; Camacho-Cruz et al. 2020). Three cenotes exceeded this level: CA (13.7 μmol/L, 0.25 mg/L), PM (102 μmol/L, 1.84 mg/L), and Y (7.90 μmol/L, 0.14 mg/L), as did the SGD (69.2 μmol/L, 1.25 mg/L). These elevated concentrations indicate contamination from sewage discharge or inadequate wastewater treatment, particularly at cenote PM, likely from outdated or insufficient wastewater treatment facilities (Moreno-Pérez et al. 2021) which are known to influence the biogeochemistry of the region’s karst water bodies (Aranda-Cirerol et al. 2011).

Ratios of dissolved inorganic nitrogen to phosphorus (DIN/P) can help identify excess nutrient loading and eutrophication and are inextricably linked to microbial growth (Smith 2006) but are also dependent on the biogeochemistry of the study area (Sterner and Elser 2003). We documented high DIN/P ratios (26–725) in the studied systems. The heightened nitrogen inputs in tandem with the co-precipitation of phosphorus and calcium carbonate in karst are likely reducing phosphorus bioavailability relative to nitrogen (Schmitter-Soto et al. 2002; Price et al. 2010), leading to phosphorus limitation, consistent with findings in cenotes (Schmitter-Soto et al. 2002) and other karst ecosystems (Maloney et al. 2011). Photosynthetic microbial growth in this study was measured using chlorophyll *a*, a universal phytoplankton pigment and common proxy for their biomass (Huot et al. 2007). Although photosynthetic microbial growth might be expected to proliferate in areas with high nutrient concentrations (e.g., cenote PM and the SGD), low light penetration or fast-moving water can limit photosynthesis and, consequently, chl *a* concentrations. Light limitation at the other studied systems (Table 1) also likely contributed to the lack of correlation between chl *a* and nutrient loading. Nonetheless, when chl *a* was detected, it occurred in open-type cenotes or the SGD, with concentrations similar to previous observations in cenotes (Schmitter-Soto et al. 2002).

Fecal coliforms and *E. coli* are key indicators of anthropogenic influence, originating from human and animal waste (Holcomb and Stewart 2020). Most of the studied systems were contaminated, and some contained *E. coli* above the U.S. EPA water quality standard (126 CFU/100 mL; U.S. EPA, 2012), three of which are used for recreation (cenotes CH, YA, and SN). The presence of fecal coliforms and *E. coli* is well-documented in wells and cenotes across the Yucatán Peninsula (Leal-Bautista et al. 2011; Borbolla-Vazquez et al. 2020; Moore et al. 2020; Arcega-Cabrera et al. 2023), and though this study represents a point in time, it is evident from the COFEPRIS data that *E. coli* contamination is a prevalent issue in cenotes located in Northern Quintana Roo (Supplemental Fig. 1). The concentrations of fecal indicator bacteria in cenotes herein (Fig. 3) were higher than those reported a decade ago (Leal-Bautista et al. 2011), a possible result of the rapid urban development in recent years, as our findings are consistent with more recent data (Borbolla-Vazquez et al. 2020; Arcega-Cabrera et al. 2023). Arcega-Cabrera et al. (2023) documented *E. coli* in cenotes (0–252 CFU/100 mL) in the northern Yucatán Peninsula. Our study surpasses their maximum observed concentration in four cenotes (PM, SN, YA, and Y), indicating greater fecal matter presence at these sites, particularly cenotes PM (1800 CFU/100 mL) and Y (613 CFU/100 mL), though season could also contribute to the variation. Arcega-Cabrera et al. (2023) sampled during the dry season, whereas our work was conducted in the rainy season and could be affected by increased bacterial loads from polluted runoff. Borbolla-Vazquez et al. (2020) found higher concentrations of fecal coliforms in cenotes located near Cancun compared to ours, likely due to more intense development in Cancun. These findings, combined with the COFEPRIS data, suggest ongoing fecal contamination in cenotes which is often attributed to inadequate wastewater infrastructure, sewage disposal, and poor wastewater effluent management along the Yucatán Peninsula.

### Contaminants of Emerging Concern (CECs)

While the presence of *E. coli* found in aquatic ecosystems raises human health concerns, the presence of antibiotic-resistant *E. coli* poses an even greater risk. Antibiotic resistance is a naturally occurring microbial defense mechanism, but it is proliferated by the release of waste laden with pharmaceuticals, antibiotic-resistant genes, or antibiotic-resistant organisms (Amarasiri et al. 2020). Aquatic ecosystems are particularly effective at facilitating the dispersal of pharmaceuticals and antibiotic resistance. The spread of pharmaceuticals fosters selective pressure of antibiotic resistance in bacterial communities, while the dispersion of AROs facilitates their contact with indigenous or human microbiota (Taylor et al. 2011; Amarasiri et al. 2020). Today, antibiotic resistance is common in Mexican waterways, partly due to insufficient regulation of antibiotic prescription(s) (Amabile-Cuevas 2021) coupled with the aforementioned inadequate waste management practices. However, a coherent picture of antibiotic resistance in Mexico is lacking because of the patchwork of scientific reports with varying methodology (Amabile-Cuevas 2021). This inconsistency is also evident in studies conducted in the coastal and karst-associated water bodies of the Yucatán Peninsula, which provide valuable insights, but are limited in number and differ in methodology from the present study (Moore et al. 2020; Guillén-Chable et al. 2022).

Previous reports have documented the presence of antibiotic-resistant genes in water bodies of the Yucatán Peninsula (Guillén-Chable et al. 2022), including cenotes (Moore et al. 2020), identifying resistant genes to a variety of antibiotic classes (e.g., ß-lactamase, macrolide, cephalosporin, fluoroquinolone) and compounds (e.g., erythromycin). Sulfamethoxazole, erythromycin, and clarithromycin are frequently detected in wastewater effluent (Omuferen et al. 2022) and widely used to treat illnesses (Wormser and Keusch 1983). Previous reports have detected resistant genes to these antibiotics in cenote microbes (e.g., Moore et al. 2020), and thus, it is unsurprising that *E. coli* in cenotes are widely resistant to these antibiotics (Fig. 4). Sulfamethoxazole was one of the first antibiotics (Wormser and Keusch 1983), and following its predominance in the 1900s, there was a spread of sulfamethoxazole-resistant organisms. The antibiotic is now combined with trimethoprim for increased effectiveness (Wormser and Keusch 1983). Future work should investigate these antibiotics in tandem to assess the efficacy of resistance to the current effective pharmaceutical treatment. Many of the *E. coli* isolates were also resistant to Penicillin G, Ceftibuten, and Chloramphenicol, which has been documented in *E. coli* inhabiting other aquatic ecosystems (Amaya et al. 2012; Odonkor and Addo 2018). It is interesting that we observed little resistance to tetracycline and levofloxacin, as multiple reports have identified antibiotic resistance or ARGs for each of these antibiotics in Mexico (Rosas et al. 2015; Delgado-Gardea et al. 2016; Guillén-Chable et al. 2022; Tapia-Arreola et al. 2022). The susceptibility to levofloxacin and tetracycline could indicate their limited use in the region compared to other antibiotics or suggests that the concentrations in the resistance assays were higher than that used in prescriptions, surpassing the *E. coli* strains’ resistance threshold.

Strains of antibiotic-resistant *E. coli* were detected in most cenotes (Fig. 4), the majority of which were resistant to five or more antibiotics. Notably, cenote PM contained more antibiotic resistance strains (10 strains) than any of the other cenotes (3 strains). This cenote also contained the highest concentrations of fecal coliforms and *E. coli* (Fig. 3). This may be because this cenote is located in a highly visited recreational park near the center of Playa Del Carmen, with an urban cover of 96%. Contrasting this cenote, fecal coliforms and *E. coli* were not present at cenote C and the SGD, so it is not surprising that there were no antibiotic-resistant *E. coli* strains at these locations. This may be a function of the limited anthropogenic influence on the sites, reflected by their moderate urban cover (18.5% and 25.5%, respectively), or for other yet unknown reasons.

Perfluoroalkyl substances are critical CECs detected worldwide (Muir and Miaz 2021; Podder et al 2021; Lyu et al. 2022); some are known to bioaccumulate (Conder et al. 2008; Casal et al. 2017; Nolen et al. 2022, 2024), persist in ecosystems (Beach et al. 2006; Ahrens and Bundschuh 2014), cause ecotoxicity (Ankley et al. 2021; Krupa et al. 2022), and disrupt microbial communities (Davis et al. 2024). PFAS concentrations in aquatic ecosystems range from low pg/L and ng/L (Muir and Miaz 2021; Podder et al. 2021) to µg/L levels at contaminated sites that are influenced by point source release(s) or adjacent to industry (Campo et al. 2015; Hepburn et al. 2019; Nolen et al. 2022). Concentrations in the studied systems ranged from 0.68 to 10.71 ng/L, relatively low compared to urban waters (Allinson et al. 2019; Meng et al. 2021) and karst ecosystems near PFAS sources (McAdoo et al. 2022). McAdoo et al. (2022) documented PFAS in groundwater fed by a karst aquifer and reported sites that contained PFAS in ranges from below the reporting level to 20 ng/L, with specific sites containing concentrations surpassing 70 ng/L, well above the levels reported in the current study. The two compounds detected in this study, PFOS and PFHxA, are commonly found in other water bodies (Hepburn et al. 2019; Podder et al. 2021; Xu et al. 2021a, b) and PFOS is frequently the most commonly detected homolog (Muir and Miaz 2021). Knowledge of PFAS contamination in Mexican waters in scarce, though a recent report (Rodríguez-Varela et al. 2021) revealed high concentrations (sum PFAS > 300 ng/L) in the wastewater of Mexico City. Mexico does not monitor PFAS in drinking water and has not proposed a restriction for their occurrence, but the U.S. EPA under the Safe Drinking Water Act has set limits on several PFAS compounds. This includes a maximum contaminant level for PFOS in drinking water set to 4 ng/L (U.S. EPA 2023). Notably, two cenotes surpassed this level, CR (7.63 ng/L PFOS) and YK (5.08 ng/L), raising concerns.

### Isotopic Hydrology

The identification and quantification of contaminants is often preceded by examining the factors that control contamination, such as whether they are locally derived or transported over long distances. Using urban cover as a metric, we sought to determine if local human development—a potential source of contamination—is correlated with contaminant levels in the studied cenotes. However, we did not observe significant correlations (Fig. 2) when evaluating the subset of systems (*n* = 8). These findings suggest that other factors govern contamination, which we postulate is primarily due to the complex geological and, consequently, hydrological characteristics of karst.

Oxygen and hydrogen stable isotopes are reliable, conservative tracers of the origin and transformation of water (Tweed et al. 2019) and are widely used to investigate recharge, mixing, and the residence time of water bodies, including in karst (Lastennet and Mudry 1997; Rusjan et al. 2019; Jang et al. 2021; Cejudo et al. 2022; Mance et al. 2022). Notably, the influence, or lack thereof, of processes that may alter isotope composition, like evaporation or mixing of water bodies, can be inferred by comparing its isotopic signature to that of meteoric water (Tweed et al. 2019). For instance, shifts in δ^2^H and δ^18^O values to the right of the Local Meteoric Water Line (LMWL) (Tweed et al. 2019) can result from evaporation preferentially removing lighter isotopes and enriching the remaining water in heavier isotopes. Consequently, comparing the studied systems based on their proximity to the LMWL and d-excess values can provide insights into the processes influencing their hydrology. Here, water bodies with rapid infiltration are suggested to closely align with the LMWL.

The isotopic compositions of the studied systems are similar to those observed across the Yucatán Peninsula (Fig. 6). Most systems’ proximity to the LMWL, combined with their high d-excess values (~ 9–14), suggests rapid cycling of meteoric water. This interpretation is further supported by significant correlations between isotope parameters (δ^2^H, δ^18^O, and d-excess) and the karstification-related ion, HCO₃⁻ (Fig. 2). The rapid infiltration of rainwater into the karst may limit water–atmosphere interactions, such as evaporation (Clark and Fritz 2013). These meteoric waters, which remain in contact with carbonate rocks, dissolve them, increasing the HCO₃⁻ concentrations (Ford and Williams 2007). The patterns reported here are consistent with findings from other karst ecosystems, where higher HCO₃⁻ concentrations were associated with high-flow and low-residence times (Lastennet and Mudry 1997), and periods of increased water–rock interactions (Jang et al. 2021).

Rapid movement of meteoric water into the karst is not surprising given the Riviera Maya, situated on the Holbox fracture zone, is characterized by high porosity and permeability, particularly near Tulum (Bauer-Gottwein et al. 2011). This is supported by the limited variability in the isotopic signature observed over three years at Rio Secreto, suggesting that the groundwater is primarily derived from regional meteoric precipitation, with minimal influence from seasonal fluctuations or evaporation, and confirming the aquifer’s fast recharge (Lases-Hernandez et al. 2019, 2020). Overall, the studied cenotes that cluster near the LMWL—particularly CH, CA, CR, and Y, which group near Rio Secreto—appear to experience short residence times and quick infiltration into the aquifer, indicating a high degree of connectivity to regional groundwater flows. Furthermore, the isotopic composition at the SGD aligned directly with the LMWL, indicating fast transport of rainwater through the aquifer to this coastal location along the typical inland-to-coast flow path of Riviera Maya groundwater (Bauer-Gottwein et al. 2011).

Whereas the lower d-excess values at cenotes S (2.6) and SN (6.1) suggest, these cenotes undergo processes that alter meteoric isotope composition. For cenote S–the only estuarine cenote sampled–isotope signatures are shifted toward that of Yucatán seawater (Socki et al. 2002; Perry et al. 2003; Lases-Hernandez 2013; Haukebo 2014), confirming that groundwater traveling inland to coast is mixing with seawater at this cenote. Notably, connections between the ocean and the carbonate aquifer are present throughout the Caribbean coast, not only in estuarine water bodies (Beddows et al. 2007). The direction of flow for groundwater or saline water depends on the complexity of the conduits and seasonal variability, further illustrating the intricate hydrology of this area (Beddows et al. 2007). For cenote SN, low d-excess suggests more evaporative conditions compared to the other systems, which may result from a longer residence time and greater exposure to insolation effects that also proliferate the growth of photosynthetic organisms at this cenote (Table 3). Similar to this cenote (SN), a minority of the groundwater data compiled for the Yucatán Peninsula exhibited low d-excess values and isotopic compositions to the right of the LMWL (Fig. 6). This is likely influenced by evaporative processes, similar to those reported for lakes in the region (Perry et al. 2003; Hodell et al. 2012; Evans et al. 2018).

The increased permeability and porosity of karst cause them to be particularly vulnerable to pollution, and they represent critical areas where contaminants can spread over large distances without being significantly filtered (Kalhor et al. 2019). The isotope compositions of the studied systems indicate that most experience short residence times, rapid recharge, and are highly connected to the regional aquifer. Therefore, it is likely that pollutants travel long distances to and from most of the studied systems, and that the fate of these contaminants is further complicated by a complex network of conduits along the coast, both of which may obscure the impact of local urban development on pollutant loading.

## Conclusion

This work contributes to the literature on contaminants in karst ecosystems located in Mexico, particularly CECs in these environments. The study assessed anthropogenic contaminants (metals, nutrients and fecal indicator bacteria) and those of emerging concern (AROs and PFAS) in ten cenotes and one SGD site along the Riviera Maya in Mexico. In addition, an effort was made to investigate the role of urban cover on contaminant loading. Contaminants were found at all sites, but significant relationships between contaminants and urban cover were not documented, a result we suggest is produced by the hydrology of karst aquifers, where interconnected water bodies exchange water through complex interactions. These findings are concerning for two major reasons: (i) cenotes are the surface expression of the underlying aquifer, and their connectivity could facilitate contaminant spread, like antibiotic resistance to microbiota over large distances or introducing harmful contaminants (e.g., PFAS and fecal indicator bacteria) into drinking water, and (ii) the popularity of cenotes as recreational sites provides a direct avenue for contaminants to affect human health, particularly those posing significant health threats (e.g., fecal coliforms and AROs). Lastly, contaminants were detected at the submarine groundwater discharge site and the estuarine cenote. Both systems are located downstream of the others and very near to the ocean, discharging regional groundwater to the Caribbean. Thus, it is likely that contaminants from anthropogenic sources travel to the Caribbean via submarine groundwater discharges and estuarine cenotes. However, due to the interconnectivity and heterogeneity of karst systems, understanding the source and fate of contaminants remains challenging, and thus, detailed studies tracing contaminants from cenotes to sites of groundwater–seawater mixing are needed.

This study offers valuable insight into the contamination of the Yucatán’s coastal karst, but we emphasize that it only captures a moment in time. Therefore, we strongly encourage continued and expanded surveillance of cenotes, including additional sites, and evaluations across seasons and varying depths. Moving forward, researchers should continue to identify and quantify contaminants in cenotes, in tandem with investigating the underlying hydrological processes that may control their presence and distribution. Given the lack of correlation between urban cover and the studied contaminants, we hypothesize that karst hydrology may be the dominant factor influencing contaminant dynamics in this area. Future efforts will be important in improving the strength of correlative analyses and confirming the role of karst hydrology in contaminant fate or help to constrain our understanding of when and how urban cover does impact contaminant loading in karst. Lastly, we encourage work that investigates the genomes of isolated-resistant bacteria, to connect phenotypic observations of multidrug resistance as observed here, to genomic mechanisms (e.g., mutations).

Comprehensive investigations of karst ecosystems are difficult to conduct, but addressing the contamination of these resources is pertinent to maintaining their groundwater quality for human uses and preserve their ecological, economical, and cultural significance. As the Yucatán Peninsula grows economically and urban development continues at an accelerated rate, plans for comprehensive water management with an emphasis on effective wastewater treatment are urgently needed to preserve the health of these ecosystems and the communities that rely on them.

## Supplementary Information

Below is the link to the electronic supplementary material.

Supplementary file1 (DOCX 2696 KB)

## Data Availability

The data generated during the current study are available from the corresponding author upon reasonable request.
