# Supplementary material for: Investigation of Anthropogenic and Emerging Contaminants in Sinkholes (Cenotes) of the Great Mayan Aquifer, Yucatán Peninsula
Source: Arch Environ Contam Toxicol. 2025 Sep 9;89(3):279–99. doi: 10.1007/s00244-025-01149-2 (PMC12568887; doi:10.1007/s00244-025-01149-2)
Supplement: Supplementary file 1 — Supplementary file1 (DOCX 2696 KB) [file 244_2025_1149_MOESM1_ESM.docx]

**Supplemental Materials**

**Investigation of anthropogenic and emerging contaminants in sinkholes (cenotes) of the Great Mayan Aquifer, Yucatán Peninsula.**

Sarah Kopczynski,^a^ Rayna Nolen,^a,b^ David Hala,^a,c^ Fernanda Lases-Hernández,^d^ Wendy Escobedo-Hinojosa,^d^ Flor Arcega-Cabrera,^d^ Ismael Oceguera-Vargas,^d^ Antonietta Quigg^a,c,e^

Corresponding author: Sarah Kopczynski

Corresponding author affiliation: Department of Marine Biology, Texas A&M University at Galveston, 200 Seawolf Parkway, Galveston, TX 77553, USA

Corresponding author email: sndavis1@tamu.edu

**Supplemental Table 1** Detection limits (DL) for analyzed metals. Strontium (Sr) was measured on a PerkinElmer AAnalyst 800 Atomic Absorption Spectrometer (PerkinElmer, Waltham, MA, U.S.) on air-acetylene flame mode and Cadmium (Cd), Nickel (Ni), and Lead (Pb) on a PerkinElmer PinAAcle900 Atomic Absorption Spectrometer (PerkinElmer, Waltham, MA, U.S.) coupled to a graphite furnace.

|  | Sr (mg/L) | Cd (ug/L) | Ni (ug/L) | Pb (ug/L) |
| --- | --- | --- | --- | --- |
| DL | 0.0003 | 0.002 | 0.0006 | 00003 |

**Supplemental Table 2** Reagents and standards for the measured metals and ions.

| **Parameter** | **Reagent / Standard** | **Supplier** | **Location** |
| --- | --- | --- | --- |
| Metals | Strontium, Pure Atomic Spectroscopy Standard | PerkinElmer | Waltham, MA, U.S. |
| Metals | Cadmium, Pure Atomic Spectroscopy Standard | PerkinElmer | Waltham, MA, U.S. |
| Metals | Nickel, Pure Atomic Spectroscopy Standard | PerkinElmer | Waltham, MA, U.S. |
| Metals | Lead, Pure Atomic Spectroscopy Standard | PerkinElmer | Waltham, MA, U.S. |
| Hardness | EDTA·2H_2_O A.C.S. | J.T.Baker | Phillipsburg, NJ, U.S. |
| Hardness | BUFFER pH 10 INCOLORO | J.T.Baker | Phillipsburg, NJ, U.S. |
| Hardness | MgCl_2_·6H_2_O A.C.S. | J.T.Baker | Phillipsburg, NJ, U.S. |
| Hardness | CaCO_3_ A.C.S. | J.T.Baker | Phillipsburg, NJ, U.S. |
| Hardness | NET A.C.S. | Meyer Lab | Blue Springs, MO, U.S. |
| Sulfates | Na_2_SO_4_ Anhydrous A.C.S. | J.T.Baker | Phillipsburg, NJ, U.S. |
| Sulfates | MgCl_2_·6H_2_O A.C.S. | J.T.Baker | Phillipsburg, NJ, U.S. |
| Sulfates | BaCl₂ A.C.S. | Meyer Lab | Blue Springs, MO, U.S. |
| Chlorides | AgNO_3_ A.C.S. | Sigma-Aldrich | St. Louis, MO, U.S. |
| Chlorides | K_2_CrO_4_ A.C.S. | J.T.Baker | Phillipsburg, NJ, U.S. |
| Chlorides | NaCl A.C.S. | J.T.Baker | Phillipsburg, NJ, U.S. |
| Alkalinity | HCl A.C.S. | J.T.Baker | Phillipsburg, NJ, U.S. |
| Alkalinity | Na_2_CO_3_ A.C.S. | J.T.Baker | Phillipsburg, NJ, U.S. |

**Supplemental Table 3** Nutrient concentrations reported in mg/L.

|  | Phosphate | Silicate | Ammonium | Nitrate | Nitrite |
| --- | --- | --- | --- | --- | --- |
| CA | 0.03 | 1.60 | 0.25 | 1.31 | 0.16 |
| CH | 0.01 | 2.41 | 0.03 | 4.94 | 0.02 |
| C | 0.02 | 1.44 | 0.05 | 7.07 | 0.05 |
| CR | 0.02 | 2.03 | 0.04 | 5.62 | 0.05 |
| PM | 0.10 | 1.53 | 1.84 | 1.40 | 0.28 |
| SN | 0.02 | 1.38 | 0.02 | 0.18 | 0.01 |
| S | 0.05 | 2.33 | 0.06 | 1.67 | 0.03 |
| YA | 0.02 | 2.18 | 0.05 | 3.91 | 0.08 |
| YK | 0.02 | 2.10 | 0.04 | 4.76 | 0.04 |
| Y | 0.06 | 2.94 | 0.14 | 25.61 | 0.11 |
| SGD | 0.01 | 1.23 | 1.25 | 0.19 | 0.01 |

**Supplemental Table 4** Media and conditions to isolate and enumerate heterotrophic aerobic bacteria, coliforms, fecal coliforms, and *E. coli*.

| Media | Temperature (ºC) | Time (hr) | Type of culture media | Selection pressure |
| --- | --- | --- | --- | --- |
| Plate Count Agar  Cat. 70152  NutriSelect®, Merk, Darmstadt, Germany | 35 ºC | 48 ± 3 | Non-selective | Heterotrophic aerobic bacterial populations.  (Greenberg et al., 1992) |
| Violet red bile agar  Cat. 70188  NutriSelect®, Merk, Darmstadt, Germany | 35 ± 0.5 ºC | 24 ± 2 to 48 ± 3 | Selective | Total coliforms.  (Eaton et al., 1998) |
| Violet red bile agar  Cat. 70188  NutriSelect®, Merk, Darmstadt, Germany | 44.5 ± 0.2 ºC | 24 ± 2 | Selective | Fecal coliforms when media is combined with the temperature used herein.  (Eaton et al., 1998) |
| ECD Chromoselect Agar with MUG  Cat. 09142  NutriSelect®, Merk, Darmstadt, Germany | 35 ± 0.5 ºC | 18 to 24 | Selective | Detects *E. coli* as blue/fluorescent colonies. (Feng & Hartman, 1992) |

**Supplemental Table 5** Details for the mass spectrometric settings used to quantify PFAS including the mass-to-charge ratio (m/z) of the monitored precursor > product ions, the fragmentor (Frag) and collision energy (CE) in voltage (V), and the compounds chromatographic retention times (R.T.) in minutes. The internal standard used for the quantification of each PFAS homolog based on the relative response ratio correction are detailed where each homolog quantified using 13C4-MPFOS and 13C8-PFOA are noted by an a and b superscript, respectively. The instrument’s limit of detection (LOD) and the extraction method percent recoveries are listed for each PFAS.

|  | Chemical Name | Molecular Formula | Precursor Ion (^m^/_z_) | Product Ion 1 (^m^/_z_) | Product Ion 2 (^m^/_z_) | Frag (V) | CE (V) | R.T. (min) | LOD | Percent Recovery |
| --- | --- | --- | --- | --- | --- | --- | --- | --- | --- | --- |
| 13C4-MPFOS | Sodium perfluoro-1-[1,2,3,4-13C4]octanesulfonate | C_4_^13^C_4_F_17_NaO_3_S | 503.00 | 80.0 | -- | 215 | 60 | 11.7 | -- | -- |
| 13C8-PFOA | Perfluoro-n-[1,2,3,4-13C8]octanoic acid | ^13^C_8_HF_15_O | 421.00 | 376.1 | 171.9 | 75 | 4 | 10.8 | -- | -- |
| PFBS^a^ | Perfluorobutane-  sulfonic acid | C_4_HF_9_O_3_S | 298.94 | 98.9 | 80.1 | 135 | 36 | 7.0 | 6.25 | 99.6 ± 24.6 |
| PFHxA^b^ | Perfluorohexanoic acid | C_6_HF_11_O_2_ | 312.97 | 269.0 | 237.3 | 100 | 8 | 8.4 | 3.125 | 91.8 ± 15.6 |
| PFHxS^a^ | Perfluorohexane sulfonate | C_6_HF_13_O_3_S | 398.93 | 98.9 | 79.9 | 195 | 45 | 9.8 | 0.39 | 110.2 ± 28.9 |
| PFHpA^b^ | Perfluoroheptanoic acid | C_7_HF_13_O_2_ | 362.97 | 319.0 | 168.8 | 85 | 0 | 9.8 | 3.125 | 93.3 ± 12.2 |
| PFOA^b^ | Perfluorooctanoic acid | C_8_HF_15_O_2_ | 412.96 | 369.0 | 354.0 | 75 | 4 | 10.8 | 6.25 | 96.1 ± 4.0 |
| PFOS^a^ | Perfluorooctane sulfonic acid | C_8_HF_17_O_3_S | 498.93 | 98.4 | 80.0 | 215 | 60 | 11.6 | 3.125 | 88.4 ± 12.0 |
| PFNA^b^ | Perfluorononanoic acid | C_9_HF_17_O_2_ | 462.96 | 419.1 | 218.9 | 85 | 4 | 11.64 | 3.125 | 96.7 ± 16.7 |
| PFDA^b^ | Perfluorodecanoic acid | C_10_HF_19_O_2_ | 512.96 | 469.0 | 269.0 | 125 | 10 | 12.4 | 6.25 | 52.7 ± 16.6 |
| PFUnA^b^ | Perfluoroundecanoic acid | C_11_HF_21_O_2_ | 562.95 | 519.0 | 269.0 | 120 | 10 | 13.0 | 3.125 | 68.5 ± 25.7 |
| PFDoA^b^ | Perfluorododecanoic acid | C_12_HF_23_O_2_ | 612.95 | 569.0 | 268.9 | 120 | 5 | 13.6 | 1.56 | 55.5 ± 16.4 |
| PFTrDA^b^ | Perfluorotridecanoic acid | C_13_HF_25_O_2_ | 662.95 | 619.0 | 269.1 | 140 | 9 | 13.9 | 3.125 | 45.0 ± 17.6 |
| PFTeDA^b^ | Perfluorotetradecanoic acid | C_14_HF_27_O_2_ | 712.94 | 668.9 | 269.2 | 140 | 10 | 14.5 | 3.125 | 77.3 ± 28.9 |

**Supplemental Table 6** Detailed transitions for the mobile phase gradient during PFAS LC-MS/MS analysis. Mobile phase A comprised Milli-Q water and mobile phase B methanol, both containing 5 mM ammonium acetate.

| Time (min) | Mobile Phase A (%) | Mobile Phase B (%) | Flow |
| --- | --- | --- | --- |
| 0.50 | 90.0 | 10.0 | 0.400 mL/min |
| 2.00 | 70.0 | 30.0 | 0.400 mL/min |
| 14.00 | 5.0 | 95.0 | 0.400 mL/min |
| 15.00 | 5.0 | 95.0 | 0.400 mL/min |
| 15.01 | 0.0 | 100.0 | 0.400 mL/min |
| 17.00 | 0.0 | 100.0 | 0.400 mL/min |
| 19.00 | 95.0 | 5.0 | 0.400 mL/min |

**Supplemental Table 7** Average PFAS concentrations (ng/L) at all sites. Standard deviation of duplicates is stated in parentheses and LOD is limit of detection. Bolded values were kept in downstream analysis because the compound of interest was detected in both duplicates. The ΣPFAS was calculated using only the concentrations where there was detection in both duplicates.

|  | PFBS | PFHxA | PFHxS | PFOS | PFDoA | ΣPFAS |
| --- | --- | --- | --- | --- | --- | --- |
| CA | <LOD | **7.15 (0.53)** | <LOD | **3.56 (3.12)** | <LOD | 10.71 (3.65) |
| CH | 0.63 (0.90) | 0.64 (0.90) | <LOD | <LOD | <LOD | <LOD |
| C | <LOD | **1.32 (0.04)** | <LOD | 0.003 (0.005) | <LOD | 1.32 (0.04) |
| CR | 0.63 (0.89) | 0.65 (0.91) | <LOD | **7.63 (1.21)** | <LOD | 7.63 (1.21) |
| PM | <LOD | <LOD | 0.41 (0.57) | **2.51 (0.92)** | <LOD | 2.51 (0.92) |
| SN | <LOD | <LOD | <LOD | **1.25 (0.47)** | <LOD | 1.25 (0.47) |
| S | 0.64 (0.90) | **1.35 (0.04)** | <LOD | **2.29 (0.00)** | <LOD | 3.64 (0.04) |
| YA | <LOD | **0.67 (0.95)** | <LOD | 0.23 (0.33) | <LOD | <LOD |
| YK | <LOD | <LOD | <LOD | **5.08 (1.93)** | 0.35 (0.50) | 5.08 (1.93) |
| Y | <LOD | 0.66 (0.94) | <LOD | **0.68 (0.00)** | <LOD | 0.68 (0.00) |
| SGD | <LOD | <LOD | <LOD | **1.54 (0.67)** | <LOD | 1.54 (0.67) |

**Supplemental Table 8** Concentrations of heterotrophic aerobic bacteria, coliforms, fecal coliforms, and *E. coli* at the sites. All bacterial concentrations are reported in colony forming units per 100 mL (CFU/100 mL). Bolded values are above the U.S. EPA’s advisory concentration (126 CFU/100 mL) for *E. coli* in recreational water bodies.

|  | Heterotrophic Aerobic Bacteria | Coliforms | Fecal Coliforms | Escherichia coli |
| --- | --- | --- | --- | --- |
| CA | 1545 | 280 | 68 | 43 |
| CH | 930 | 300 | 212 | **167** |
| C | 1004 | 0 | 0 | 0 |
| CR | 666 | 256 | 110 | 34 |
| PM | 8889 | 6667 | 5432 | **1800** |
| SN | 3000 | 2540 | 943 | **385** |
| S | 1360 | 210 | 57 | 21 |
| YA | 3429 | 1874 | 753 | **365** |
| YK | 1644 | 805 | 324 | 56 |
| Y | 4000 | 2667 | 1278 | **613** |
| SGD | 1587 | 193 | 27 | 0 |


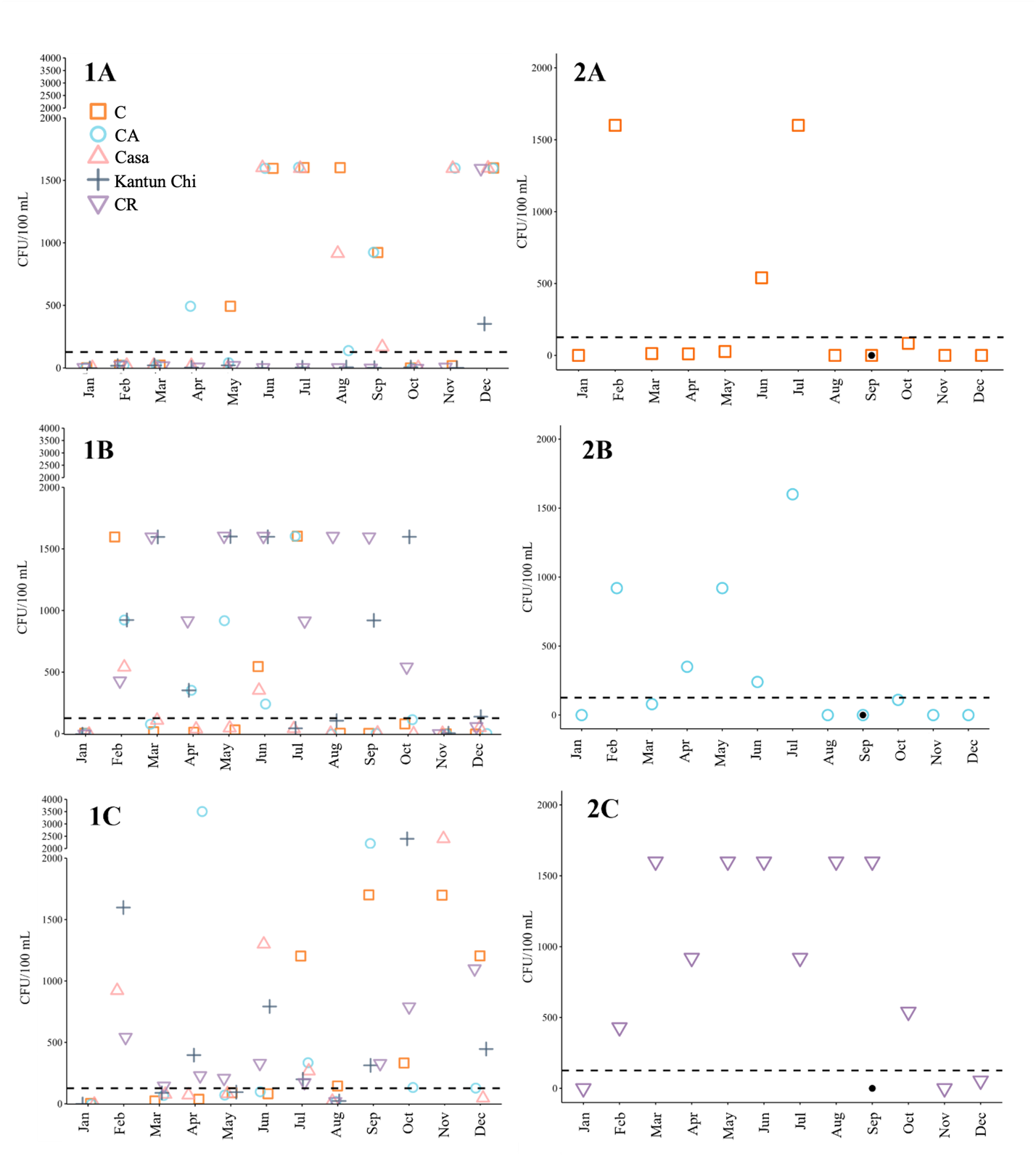


**Supplemental Fig. 1** *E. coli* concentrations (CFU/100 mL) in selected Quintana Roo cenotes measured in 2021 (1A), 2022 (1B), and 2023 (1C) by COFEPRIS. *E. coli* concentrations in cenotes C (2A), CA (2B), and CR (2C) during 2022. Data collected in the present study is documented as a circle (2). Any values above 5000 CFU/100 mL were omitted for visualization but listed here for the cenotes of interest: C, CA, and CR had *E. coli* levels above 5000 CFU/100 mL in April 2021 (16,000 CFU/100 mL), November 2023 (16,000 CFU/100 mL), and November 2023 (9,200 CFU/100 mL), respectively.


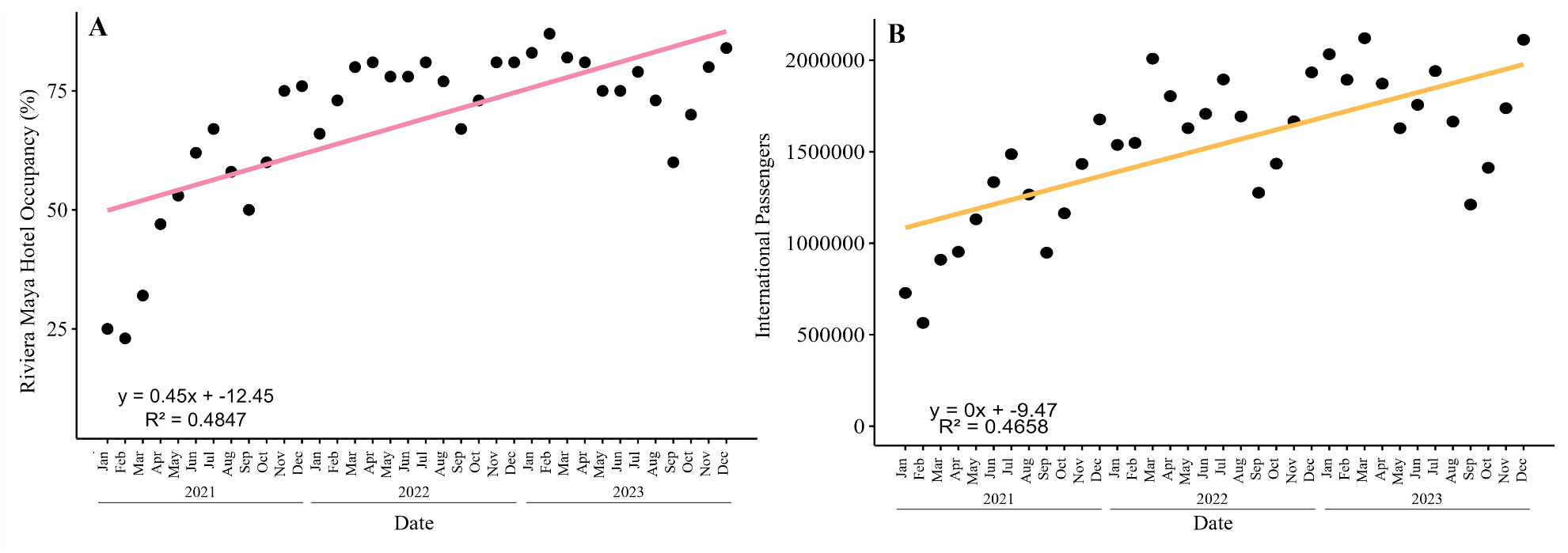


**Supplemental Fig. 2** Riviera Maya hotel occupancy (%; A) and number of international passengers flying into Quintana Roo airports (B) documented by Quintana Roo SEDETUR, with trendlines fitted to a standard linear model in R. Hotel Occupancy and international passenger numbers are cyclic, with lower numbers in the fall/winter and higher in the spring/summer. The trend lines show the overall increase post-COVID and with the continued growth of tourism in the region.
